# A driver role for GABA metabolism in controlling stem and proliferative cell state through GHB production in glioma

**DOI:** 10.1007/s00401-016-1659-5

**Published:** 2016-12-28

**Authors:** Elias A. El-Habr, Luiz G. Dubois, Fanny Burel-Vandenbos, Alexandra Bogeas, Joanna Lipecka, Laurent Turchi, François-Xavier Lejeune, Paulo Lucas Cerqueira Coehlo, Tomohiro Yamaki, Bryan M. Wittmann, Mohamed Fareh, Emna Mahfoudhi, Maxime Janin, Ashwin Narayanan, Ghislaine Morvan-Dubois, Charlotte Schmitt, Maité Verreault, Lisa Oliver, Ariane Sharif, Johan Pallud, Bertrand Devaux, Stéphanie Puget, Penelope Korkolopoulou, Pascale Varlet, Chris Ottolenghi, Isabelle Plo, Vivaldo Moura-Neto, Thierry Virolle, Hervé Chneiweiss, Marie-Pierre Junier

**Affiliations:** 1 CNRS UMR8246, Inserm U1130, UPMC, Neuroscience Paris Seine-IBPS, Sorbonne Universities, 75005 Paris, France; 2 Instituto Estadual do Cérebro Paulo Niemeyer, Secretaria de Estado de Saúde do Rio de Janeiro/RJ, Rio De Janeiro, CEP 20231-092 Brazil; 3 Université de Nice-Sophia Antipolis, Institut de Biologie Valrose, CNRS UMR7277, Inserm U1091, Nice, France; 4 Laboratoire Central d’Anatomie Pathologique, Hôpital Pasteur, University of Nice-Sophia-Antipolis, Nice, France; 5 The CPN Proteomics Facility-3P5, Inserm, U894, Paris Descartes University, 75014 Paris, France; 6 CNRS UMR 8256, Laboratory of Neuronal Cell Biology and Pathology, UPMC, Sorbonne Universities, Paris, France; 7 Metabolon, Inc., Durham, NC 27713 USA; 8 Inserm U1009, Institut Gustave Roussy, 94800 Villejuif, France; 9 Reference Center of Inherited Metabolic Diseases, University Paris Descartes, Hospital Necker Enfants Malades, APHP, Paris, France; 10 Department of Biochemistry, University Paris Descartes, Hospital Necker Enfants Malades, 75015 Paris, France; 11 Inserm U 1127, CNRS UMR 7225, Sorbonne Universités, UPMC UMR S 1127, Institut du Cerveau et de la Moelle épinière, ICM, 75013 Paris, France; 12 Team 9 CRCNA, UMR 892 Inserm, 6299 CNRS, Université de Nantes, 44035 Nantes Cedex 07, France; 13 Inserm, Jean-Pierre Aubert Research Center, Development and Plasticity of the Neuroendocrine Brain, Unit 1172, France, UDSL, School of Medicine, Place de Verdun, 59045 Lille Cedex, France; 14 Department of Neurosurgery, Sainte-Anne Hospital, Paris Descartes University, 75014 Paris, France; 15 Department of Neurosurgery, Necker-Enfants Malades Hospital, 75015 Paris, France; 16 Department of Pathology, National and Capodistrian University of Athens, Athens, Greece; 17 Department of Neuropathology, Sainte-Anne Hospital, Paris Descartes University, Paris, France; 18 Department of Bionanoscience, Kavli Institute of Nanoscience, Delft University of Technology, Lorentzweg 1, 2628 CJ Delft, The Netherlands

**Keywords:** Brain cancer, DIPG, Cancer stem cell, *ALDH5A1*, GABA, 5-hmC, Valproate

## Abstract

Cell populations with differing proliferative, stem-like and tumorigenic states co-exist in most tumors and especially malignant gliomas. Whether metabolic variations can drive this heterogeneity by controlling dynamic changes in cell states is unknown. Metabolite profiling of human adult glioblastoma stem-like cells upon loss of their tumorigenicity revealed a switch in the catabolism of the GABA neurotransmitter toward enhanced production and secretion of its by-product GHB (4-hydroxybutyrate). This switch was driven by succinic semialdehyde dehydrogenase (SSADH) downregulation. Enhancing GHB levels via SSADH downregulation or GHB supplementation triggered cell conversion into a less aggressive phenotypic state. GHB affected adult glioblastoma cells with varying molecular profiles, along with cells from pediatric pontine gliomas. In all cell types, GHB acted by inhibiting α-ketoglutarate-dependent Ten–eleven Translocations (TET) activity, resulting in decreased levels of the 5-hydroxymethylcytosine epigenetic mark. In patients, low SSADH expression was correlated with high GHB/α-ketoglutarate ratios, and distinguished weakly proliferative/differentiated glioblastoma territories from proliferative/non-differentiated territories. Our findings support an active participation of metabolic variations in the genesis of tumor heterogeneity.

**Electronic supplementary material:**

The online version of this article (doi:10.1007/s00401-016-1659-5) contains supplementary material, which is available to authorized users.

## Introduction

Tumor development is a complex process mixing clonal selection and dynamic changes in cell states including phenotypic differentiation of cancer stem cells, which leads to tumors composed of heterogeneous cancer cell populations [20, 21]. De novo glioblastoma (GBM), the most common and malignant primary brain tumor in adults, is a paradigmatic example of heterogeneous tumors. This malignant glioma remains incurable, with all patients relapsing despite aggressive multimodal therapies [43]. Its heterogeneity is illustrated by the coexistence of territories enriched in weakly or actively proliferative cells [10], and of cells with variable expression of molecular markers, differences in morphological features of differentiation, and variable tumorigenicity [37, 40]. Recent single cell genomic and transcriptomic analysis further documented this heterogeneity [37, 41]. However, the role of metabolism in the genesis of tumor cells and/or territories with differing states of aggressiveness remains unexplored. Following the recent discovery that the differentiation of embryonic stem cells (ESC) depends on fluctuations in the levels of the metabolite α-ketoglutarate (α-KG) [8], we envisaged that changes in metabolism could drive cancer cell phenotypic differentiation rather than be a passive adaptation to differentiation.

To address this issue, we focused on GBM stem-like cells, which share with ESC transcription factors such as Nanog that govern their behavior [9]. These cancer cells, endowed with self-renewal, differentiation, tumor-initiating properties and resistance to current therapies, are active drivers of tumor growth [9]. Importantly, they can oscillate between a non-differentiated, aggressive state and a differentiated, less aggressive state in response to environmental cues [5, 39].

We exploited first our recent discovery that differentiated, weakly proliferative GBM cells express the micro-RNA cluster miR-302-367, the expression of which triggers GBM stem-like cell exit from their stem and tumorigenic state [15]. This cell model served as a starting paradigm to pinpoint metabolic changes of potential relevance and to identify their molecular source. Metabolome profiling revealed an unexpected increase in the GABA by-product GHB (4-hydroxybutyrate), which we discovered to be caused by downregulation of the mitochondrial enzyme SSADH. We then determined whether increasing GHB levels might be sufficient per se to alter the cell properties, using additional and independent GBM and deep infiltrating glioma (DIPG) cells. Combining metabolite measurements, genomic and pharmacological manipulations, in vivo experiments, bioinformatics analyses of independent GBM datasets, and analysis of patients’ tumor tissues, we discovered that fluctuation in the levels of GHB suffices to switch malignant glioma cell from a proliferative and aggressive behavior to a more differentiated and less aggressive state.

## Materials and methods

All the figures were prepared using Adobe Illustrator (Adobe Systems).

### Human tissues

Glioblastoma samples from adult patients were obtained from surgical resections. For metabolite measurements and immunohistochemical analysis, glioblastoma fragments were subjected to multisampling.

### Cell culture

GBM stem-like cells TG1, TG16, GBM-M, R633 cells were isolated from neurosurgical biopsy samples of human GBM (Table S1), and their stem-like and tumor-initiating properties characterized as previously reported [4, 42, 49, 53]. TG1-miR was derived from TG1 as described [15]. GBM stem-like cells 6240**, stably expressing a luciferase construct, GBM stem-like cells 5706**, and JolMa cells were characterized as described [7, 46]. No mutation in *IDH1* or *IDH2* coding regions was found (Table S1). TP54, TP80, TP83, TP84 stem-like cells with a K27M *H3F3A* mutation [58], were isolated from pediatric DIPG and characterized as previously described [52]. Molecular profiles were obtained with transcriptome analysis using Affymetrix Exon 1.0S array (3 independent biological replicates), and proneural, classical or mesenchymal subtype determined with respect to the classification of the TCGA established with a 840 genes list [55]. UT7 leukemia cell line was transduced with lentiviral vector encoding doxycycline-inducible human TET2-GFP cDNA (Fig. S6E). TG1 stem-like cells were transduced with lentiviral vectors encoding doxycycline-inducible human wild-type or catalytically deficient form of TET2-GFP cDNA (Fig. S6F). TG1, 6240**, 5706** and TP54 stem-like cells were transduced with lentiviral vectors encoding a control or an *ALDH5A1* shRNA construct (GeneCopeia, Tebu, France). In relevant experiments, cells were treated with GHB or valproate (both from Sigma) or their vehicles (cell medium).

### Metabolite measurement by mass spectrometry (MS)

Cells and media were harvested 96 h post-seeding (cell half-doubling time = 4.5, TG1, and 8 days, TG1-miR). Cell pellets were washed in PBS before freezing. Media and cell samples (*n* = 6) were extracted and analyzed on the GC/MS and LC/MS/MS platforms of Metabolon, Inc. (Durham, NC, USA) as previously described [45]. Following normalization to total protein values for cell data (Bradford assay), log transformation, and imputation with minimum observed values for each compound, Welch’s two-sample *t* tests were used to identify metabolites that differed significantly between experimental groups. The level of significance was set at *p* < 0.05. Each metabolite was mapped to pathways based on the Kyoto Encyclopedia of Genes and Genomes (KEGG) (http://www.genome.jp/kegg/ *‎)*, the human metabolome database (http://www.hmdb.ca), and literature mining. Targeted analyses of GHB (4-hydroxybutyrate) and 2-HG (2-hydroxyglutarate) were performed with GC–MS/MS (300MS, Brüker) in the clinical chemistry laboratory at Necker Enfants Malades Hospital (Paris, France). For metabolite measurements in tissues, glioblastoma fragments from adult patients were subjected to multisampling. Each sample was divided into two mirror pieces: one snap-frozen, the second reserved for immunohistological characterization. Tissue punches from weakly proliferative/differentiated (P^LOW^/D^+^) and proliferative/non-differentiated (P^HIGH^/D^−^) glioblastoma territories were obtained from the snap-frozen pieces.

### Cell death evaluation

Cell death was evaluated using the propidium iodide (PI) exclusion test. The cells were incubated with PI (10 µg/10^6^ cells) for 10 min at 4 °C, and the percentage of cells containing PI was measured using FACS (ARIA II, BD Biosciences, France).

### Clonality and self-renewal evaluation

Cells were plated at one cell/well (Nunc, 96-deep well plate, non-treated), and treated with 10 mM of GHB each 48 h over four weeks. The cells were then dissociated, and seeded at one cell/well. The percentage of wells containing spheres was scored. At least 500 cells were analyzed for each culture. For extreme limiting dilution assays (ELDA), cells were plated in 96-well plates at 1, 5, 10, 20, 50, and 100 cells/well/100 μl as previously described [4]. The percentage of wells with neurospheres was determined after 10 and 21 days. The analysis of the frequency of sphere-forming cells, a surrogate property of brain cancer stem-like cells [18] was performed with software available at http://bioinf.wehi.edu.au/software/elda/ [25].

### Cell cycle analysis

Cells were incubated with BrdU (5-Bromo-2′-deoxyuridine, 10 µM, Invitrogen) for 3 h, and analyzed after DAPI staining using an ARIA II (BD Biosciences, France). Analysis was performed on 10,000-gated events. Violet laser (405 nm) and Pacific Blue filter were used for DAPI detection, and Red laser (640 nm) and APC filter for BrdU detection. Data analysis and figure generation were performed using the FACS Diva version 6.1.2 program (BD Biosciences, France).

### Cell adherence assay

Cells were plated in 96-well plates (BD Biosciences, BD BioCoat Poly-d-Lysine) at a density of 5 × 10^3^ cells/well. The number of adherent cells was counted after 24 h of incubation and expressed in percentage of the total cell numbers. Images were acquired on a digital camera (DXM 1200, Nikon, USA) using AxioVision 4.6 Software (Laboratory Imaging, Ltd).

### siRNA transfection

Cell transfection was achieved with the Amaxa Nucleofector Electroporator using the Nucleofector program A-020 (Amaxa Biosystems, Gaithersburg, MD, USA). Cells were transfected by electroporation with 1 μM of control siRNA (Ambion^®^ Silencer Negative Control, Cat#AM4611), or anti-*ALDH5A1* siRNAs (Ambion^®^ Cat#16,708, ID si15460, Cat#16,708 ID si15462), or anti-TET2 siRNAs (Ambion^®^ Cat#4392420, ID si29443). The transfection was performed using the L transfection solution (AMAXA). The cells were chocked twice (at day 0 and day 3) and collected at day 6.

### Luciferase reporter assays

Cells were transfected with Renilla Luciferase mRNA and Firefly luciferase mRNA containing either the wild-type form of *ALDH5A1*-3′UTR or a mutant form of *ALDH5A1*-3′UTR with a deletion of the miR-302 putative target sequence (*ALDH5A1*-3′UTR-DEL). The quantification of Renilla and Firefly luciferase activity was performed using the Dual-Luciferase Reporter Assay System (Promega, France), according to manufacturer’s instructions. Renilla luciferase was used for internal normalization of Firefly activity values.

### Immunoblotting

Cells were harvested, washed with PBS and cell lysis was performed in 50 mM Tris–HCl pH 7.4 buffer containing 1% Triton X-100, 150 mM NaCl, 0.5 mM EGTA, 0.5 mM EDTA and anti-protease cocktail (Complete Protease inhibitor Cocktail Tablets, Roche, France). Protein extracts (30 μg) were separated by SDS-PAGE and transferred to Hybond-C Extra nitrocellulose membranes (GE Healthcare, USA). The following antibodies were used for immunoblotting: anti-Nanog (Cell Signaling, 1:1000), anti-p21 (Santa Cruz Biotechnology, 1:200), anti-Actin (Millipore Chemicon, 1:10,000), anti-SSAR (Novus biological, 1:2000), anti-ABAT1 (GABA-T) (Sigma Aldrich, 1:2500), anti-GAD65 (Abcam, 1:500), anti-GAD67 (Abcam, 1:500), anti-HOT (Sigma, 1:2000), anti-GLUD1 (Sigma, 1:2000), and anti-SSADH (Novus Biological, 1:2000). The secondary antibodies were anti-mouse IgG (Santa Cruz Biotechnology, 1:10,000), anti-rabbit IgG (GE Healthcare, 1:10,000) and anti-goat IgG (Santa Cruz Biotechnology, 1:10,000). Signal detection was performed with the ECL + chemiluminescence detection system (PerkinElmer, France). Densitometric analysis was achieved using ImageJ software.

### Immunocytochemistry

Cells were harvested, PBS washed, smeared on SuperFrost slides (Fischer Scientific, France), and fixed in ice cold methanol for 20 min at −20 °C. Following fixation, cells were washed with PBS, and incubated for 30 min. at room temperature in PBS containing 0.3% Triton X-100 and 5% BSA (Sigma). The following primary antibodies were incubated overnight at 4 °C: Nanog (1:200, R&D), Olig2 (1:200, R&D), GFAP (1:5000, Dako), GLT-1/EEAT2 (1:200, Santa Cruz), ß3-Tubulin (1:3000, Covance), Map-2 (1:1000, Millipore). Secondary antibodies were incubated at room temperature for 1 h (Alexa Fluor^®^ 488, Alexa Fluor^®^ 555, Molecular Probes, 1:2000). Immunostaining was analyzed with a fluorescent microscope equipped with an ApoTome module (Axioplan 2, Zeiss). Images were acquired on a digital camera using AxioVision 4.6 Software (Laboratory Imaging, Ltd) and prepared using Adobe Photoshop software (Adobe Systems, San Jose, CA). Immunofluorescent signals were analyzed with Volocity 3D Image Analysis software (PerkinElmer, France) using single optical 240 nm sections.

### Immunohistochemistry

Morphologic examination was performed on Hematoxylin- and Eosin-stained sections (3–4 μm). Immunolabeling was performed using an automated system (Autostainer Dako, Glostrup Denmark) with the following primary antibodies: anti-Ki67 (MIB-1, Dako, prediluted), anti-Olig2 (goat polyclonal, R&D Systems, 1:500) and anti-GFAP (Dako, 1:4000). Deparaffinization, rehydration and antigen retrieval were performed using the pretreatment module PTlink (Dako). SSADH immunostaining was achieved following overnight incubation with anti-SSADH (Novus Biological, 1:100) at 4 °C. Immunostaining was scored by a pathologist (FBV).

### DNA sequencing, q-PCR

RNA was extracted from the cells with RNeasy Mini Kit (Qiagen, Hilden, Germany, http://www.qiagen.com), submitted to an on-column DNase digestion (RNase-Free DNase Set, Qiagen) and retrotranscribed (QuantiTect Reverse Transcription Kit, Qiagen). PCR was performed using Platinum^®^ Taq DNA Polymerase High Fidelity (Invitrogen). The PCR products were purified and sequenced (Biofidal, Vaulx en Velin, France, http://biofidal.com). Q-PCR assays were performed using an ABI Prism 7700 Sequence Detection system (Perkin-Elmer Applied Biosystems), and the SYBR Green PCR Core Reagents kit (Perkin-Elmer Applied Biosystems). Transcripts of the TBP gene encoding the TATA box-binding protein were used for normalization.

### 5-hydroxymethylcytosine (5-hmC) and 5-methylcytosine (5-mC) detection

5-hmC and 5-mC were quantified using dot blot assays with anti-5-hmC antibody (Active Motif, Carlsbad, CA, 1:10,000) and anti-5-mC antibody (Millipore Calbiochem, Darmstadt, Germany, 1:2000). DNA was spotted onto nylon Hybond N+ 53 membranes (Amersham) and fixed by U.V. cross-linking. Membranes were air-dried, blocked and incubated with anti-5-hmC or anti-5-mC antibodies and horseradish peroxidase-conjugated secondary antibody (anti-rabbit IgG 1:10,000, GE Healthcare, anti-mouse IgG 1:10,000, Santa Cruz Biotechnology). Signal detection was performed with the ECL + chemiluminescence detection system (PerkinElmer, France). Densitometric analysis was achieved using ImageJ software.

### Intracranial xenografts

6240** GBM stem-like cells transduced with a luciferase-encoding lentivirus, 5706** GBM stem-like cells and TP54 DIPG stem-like cells stably expressing GFP were used. 100,000 (6240**), 50,000 (5706**) and 25,000 (TP54) cells were injected stereotaxically into the striatum of anesthetized 8- to 9-week-old Nude mice (Harlan Laboratories), using the following coordinates: 0 mm posterior and 2.5 mm lateral to the bregma, and 3 mm deep with respect to the surface of the skull. 16 mice were grafted with 6240** cells transduced with a shControl construct and 15 mice with a sh*ALDH5A1* construct. Luminescent imaging was performed on an IVIS Spectrum (Perkin-Elmer), after intra-peritoneal injection of luciferin. Total flux (photons per second) values were obtained by imaging mice 14 and 49 days after stereotaxic cell injection and quantified with Live Image 4.0 software. Xenografts of GFP-expressing 5706** and TP54 transduced with a shControl construct or a sh*ALDH5A1* construct were each performed into 3 (5706**) or 4 (TP54) mice per group. Mice were sacrificed at 64 (5706**) or 71 (TP54) days post-graft, and the numbers of GFP-expressing cells determined. The animal maintenance, handling, surveillance, and experimentation were performed in accordance with and approval from the Comité d’éthique en expérimentation animale Charles Darwin N°5 (Protocol #3113).

### Statistical analysis

Statistical analyses were done with Prism 6.0 software (GraphPad) using unpaired *t* test with Welch’s correction, or one-sample *t* test when appropriate unless otherwise indicated. Significance threshold was set at *p* < 0.05. Mean ± SD are shown. *p < 0.05, **p < 0.01, ***p < 0.001, ****p < 0.0001.

## Results

### GBM stem-like cells differentiation is accompanied by *ALDH5A1* downregulation, which reprograms GABA metabolism toward enhanced GHB production

Metabolic rearrangements in differentiated GBM stem-like cells were investigated using unbiased global metabolomic profiling of the TG1 cell line, which was isolated from an* IDH1* and* 2* wild-type primary GBM (Table S1). We compared naïve cells and cells stably expressing miR-302-367 (referred to as TG1-miR) that are deprived of stem and tumorigenic properties [15], and enriched in differentiation markers (see [15] and Fig. S1). Gas chromatography/mass spectrometry (GC/MS) and liquid chromatography/MS/MS analysis of whole cell extracts and secreted culture media showed that all identified metabolic intermediates and endpoint products of energy metabolic pathways, i.e., glycolysis, tricarboxylic acid (TCA) cycle, and anaplerotic glutaminolysis were significantly reduced in TG1-miR, as exemplified by α-KG a key metabolite of the TCA cycle that can be replenished through anaplerotic reactions (Table S2).

**Fig. 1:**
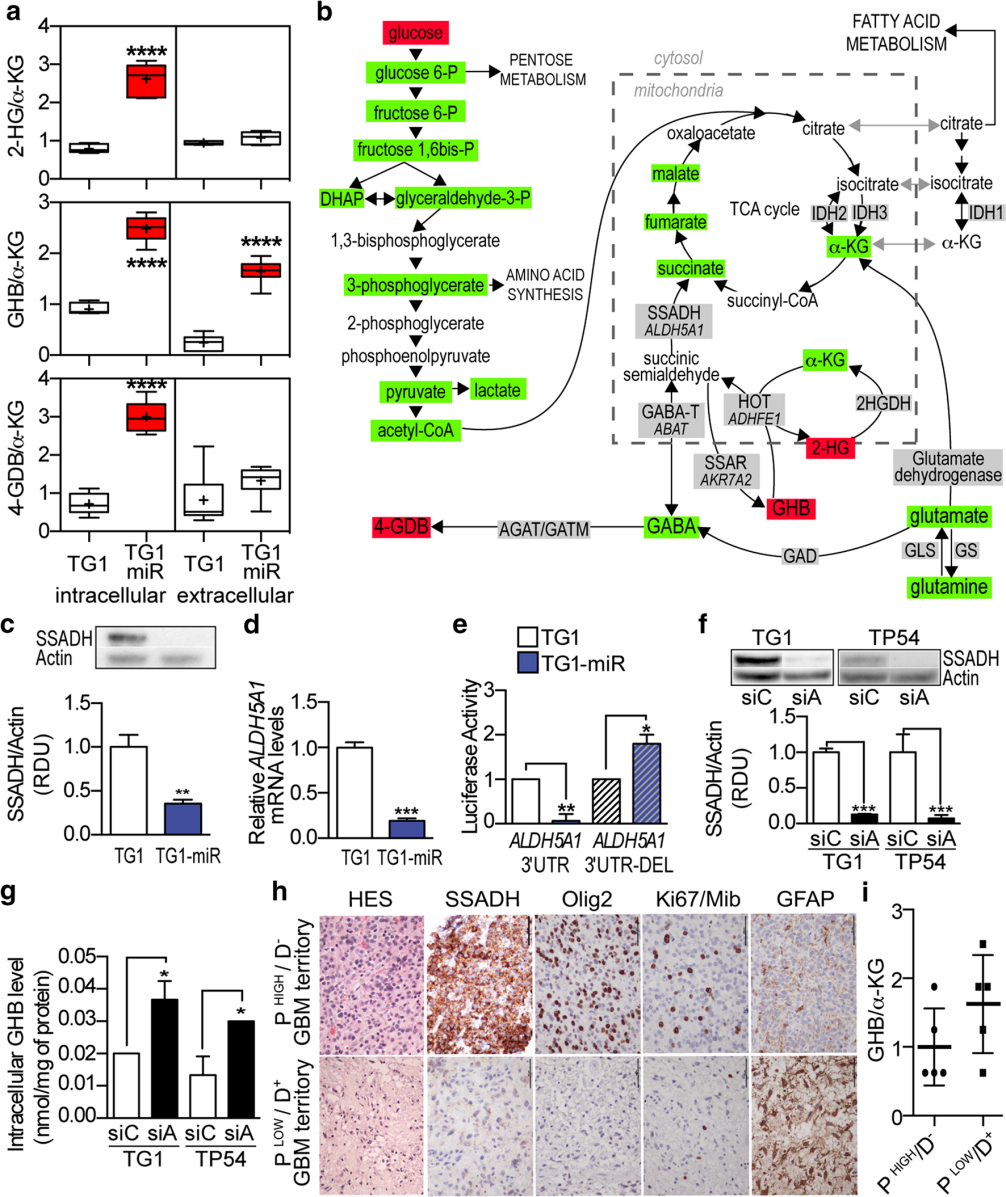
Loss of stem and tumorigenic properties by GBM stem-like cells is accompanied with GABA metabolism deregulation characterized by enhanced GHB levels. **a** Increased GABA by-products to α-KG ratios in TG1-miR compared to TG1. The “+” sign represents the mean value in the *whisker box*. Mean ± SD, *n* = 6 independent biological samples. **b** Schematic reconstruction of metabolic pathways with *green* and *red boxes* signaling metabolites decreased or increased in TG1-miR compared to TG1, respectively. Enzyme names are within *grey boxes*. When relevant, the corresponding gene designation is indicated below the enzyme name. SSADH: succinic semialdehyde dehydrogenase. SSAR succinic semialdehyde reductase. **c** Downregulation of the *ALDH5A1* protein product SSADH in TG1-miR. Western blot analysis. SSADH MW, 57 kDa; Actin MW, 42 kDa. Mean ± SD, *n* = 3 independent biological samples. **d** Decreased *ALDH5A1* mRNA levels in TG1-miR compared to TG1. Q-PCR assays. Mean ± SD, *n* = 3 independent biological samples. **e** Targeting of the *ALDH5A1* transcript by miR-302. Expression of Renilla Luciferase mRNA containing the wild-type form of *ALDH5A1*-3′UTR is strongly reduced in TG1-miR compared to TG1. Deletion of miR-302 putative target sequence in the 3′UTR of *ALDH5A1* mRNA (*ALDH5A1*-3′UTR-DEL) prevents the binding of the miR, and rescues luciferase activity. *n* = 3 independent biological samples. **f** Decreased SSADH levels in GBM and DIPG stem-like cells (TG1, TP54) upon *ALDH5A1* downregulation with siRNAs (siA). siC (control siRNA). Mean ± SD, *n* = 3 independent biological samples. RDU, relative densitometry units. **g** *ALDH5A1* down regulation results in enhanced GHB intra-cellular levels. Mean ± SD, *n* = 3 independent biological samples. **h** SSADH immunoreactive cells are enriched in proliferative/non-differentiated GBM territories (*P* ^HIGH^/*D* ^−^) and rare in non-proliferative/differentiated (*P* ^LOW^/*D* ^+^) tumor territories of patients’ GBM, as revealed by immunohistochemical staining of Ki67, Olig2, and GFAP. HES: hematoxylin and eosin staining. *Scale bar* 100 µm. **i** GHB/α-KG ratios in proliferative/non-differentiated (*P* ^HIGH^/*D* ^−^) and weakly proliferative/differentiated territories (*P* ^LOW^/*D* ^+^) of patient GBM. GC–MS/MS analysis. Mean ± SD, *n* = 5 independent patient’s GBM neurosurgical samples

This overall reduction in TG1 energy metabolism upon loss of their stem and tumorigenic properties was accompanied by a broad deregulation of GABA neurotransmitter metabolism (Fig. 1a, b). Decreased GABA levels were associated with increased levels of its metabolic by-products GHB, 2-hydroxyglutarate (2-HG), and 4-guanidinobutanoate (4-GDB) (Table S2). As a result, GABA by-products to α-KG ratios were increased in TG1-miR (Fig. 1a). Since GHB levels were increased in both intra- and extra-cellular compartments, we further focused on understanding the cause for the elevated production of GHB. As depicted in Fig. 1b, glutamate is the entry point of GABA synthesis pathway. It can either be converted into α-KG by glutamate dehydrogenase (or aminotransferases), or into GABA by glutamate decarboxylases (GAD67 and GAD65). GABA transaminase (GABA-T) catalyzes GABA conversion into succinic semialdehyde. The succinic semialdehyde reductase (SSA reductase/SSAR) is responsible for the synthesis of GHB from succinic semialdehyde, whereas hydroxyacid-oxoacid transhydrogenase (HOT) is responsible for GHB degradation. HOT catalyzes a concomitant reaction that additionally produces succinic semialdehyde and 2-HG from α-KG. *In silico* analysis identified putative miR-302-367 recognition sites in the mRNA of three enzymes of this pathway, i.e., GABA transaminase/GABA-T, hydroxy-oxoacid transhydrogenase/HOT, succinic semialdehyde dehydrogenase/SSADH (Fig. S2A). Only SSADH protein levels were markedly reduced in TG1-miR compared to TG1 (Fig. 1c, Fig. S2B). The transcript levels of *ALDH5A1,* which encodes SSADH, were also strongly reduced (Fig. 1d). To confirm the direct targeting of the 3′ UTR region of *ALDH5A1* mRNA by miR-302, we employed TG1-miR and TG1 cells expressing a Renilla luciferase expression construct containing the *ALDH5A1*-3′UTR. Luciferase activity was strongly reduced in TG1-miR compared to TG1 (Fig. 1e). Deletion of miR-302 putative target sequence in the 3′UTR of *ALDH5A1* mRNA (*ALDH5A1*-3′UTR-DEL) prevented the binding of the miR, and rescued luciferase activity (Fig. 1e). SSADH converts succinic semialdehyde into succinate, fuelling thus the TCA cycle and thereby limiting the amounts of substrate for SSAR, the enzyme responsible for GHB synthesis (Fig. 1b). Since SSAR levels were unchanged in TG1-miR (Fig. S2B), we verified whether reduced SSADH expression was sufficient to increase GHB levels. SSADH downregulation by siRNA targeting of *ALDH5A1* mRNA (Fig. 1f) caused accumulation of GHB not only in stem-like cells of adult GBM but also in stem-like cells of infant DIPG, a form of high-grade glioma with a poor prognosis [58] (Fig. 1g). No change was observed in the levels of 2-HG, the other GABA by-product (Fig. S3). To further test the link between *ALDH5A1* downregulation and enhanced GHB production, we used valproate to inhibit SSADH activity. Valproate has long been known to inhibit SSADH activity [54, 57], before being found to also inhibit histone deacetylases [19]. The adult GBM 6240** and DIPG TP54 cell lines were treated with 5 mM valproate. Mass spectrometry measurements showed a five- to sixfold increase in GHB intra-cellular levels in valproate-treated cells compared to control cells (Fig. S4). Altogether, these results show that decreased SSADH expression is sufficient to increase GHB levels.

Using neurosurgical samples of patient tumors, we then compared SSADH expression and GHB/α-KG ratios in GBM territories characterized by low or high proliferative and differentiation features. Coherently to our in vitro observations, we found that SSADH staining prevailed in proliferative/non-differentiated GBM territories (P^HIGH^/D^−^) containing mitotic and Olig2 + tumor cells (Fig. 1h, upper panels and Fig. S5A). On the opposite, SSADH expression was scant in non-proliferative/differentiated GBM territories (P^LOW^/D^+^) characterized by rare mitotic cells and lack of Olig2 expression (Fig. 1h, lower panels and Fig. S5A). Interestingly, SSADH was also detected in normal cells albeit at a lower intensity than in tumor cells of proliferative/non-differentiated GBM territories (Fig. S5B). Of note, destruction of the normal underlying axonal network distinguishing the solid tumor tissue from infiltrative peri-tumoral tissue was confirmed using neurofilament staining (Fig. S5C). In addition, GC–MS/MS analysis showed that GHB/α-KG ratios tended to be higher in these GBM territories characterized by rare mitotic cells (Fig. 1i). Altogether, these results support the pathological relevance of variations in GHB levels in the context of human GBM.

### GHB inhibits proliferation of GBM and DIPG stem-like cells

**Fig. 2:**
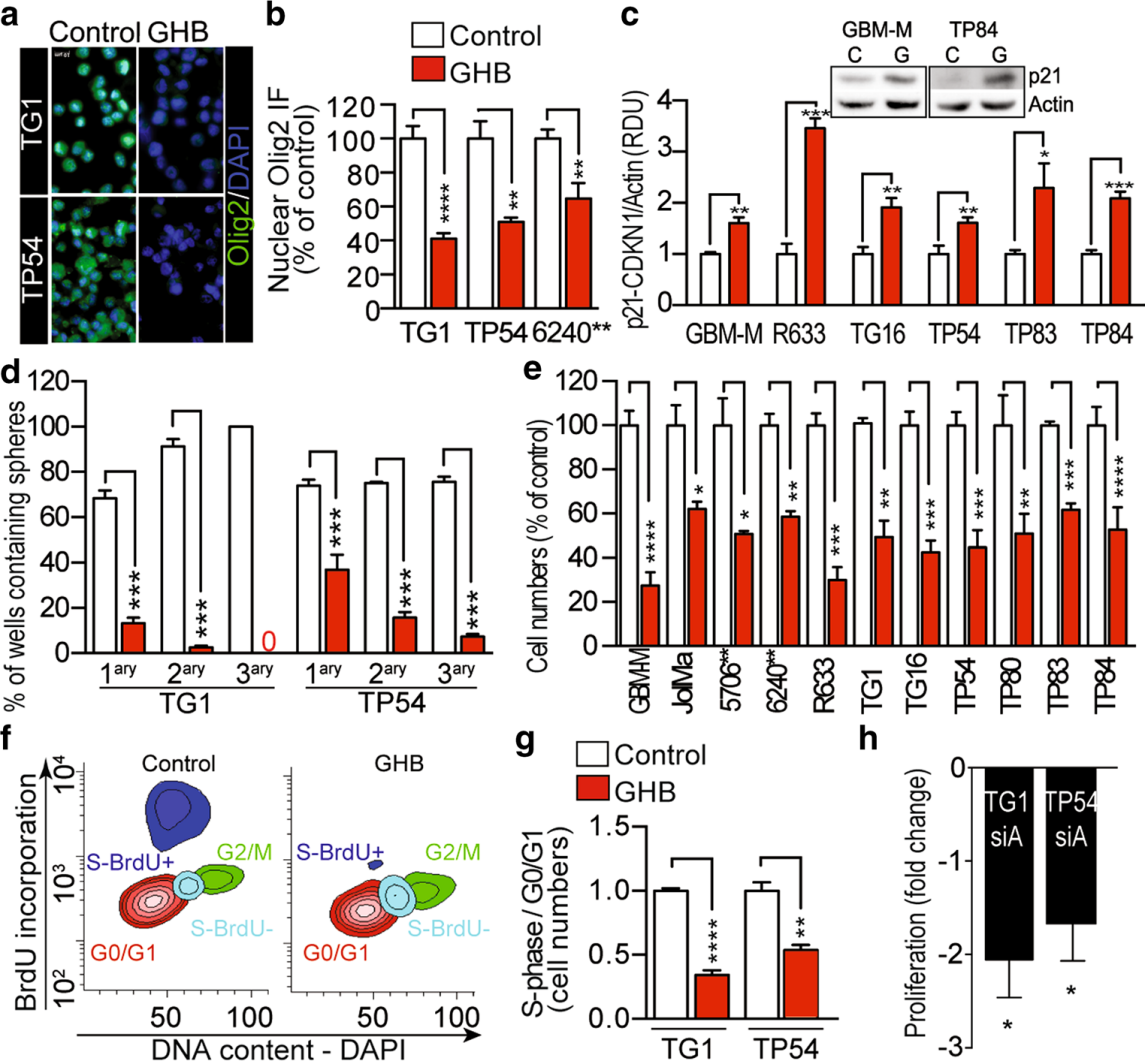
GHB inhibits GBM and DIPG stem-like cell proliferation and self-renewal. **a** Immunocytochemical detection of Olig2. 1-week GHB 10 mM. *Scale bars* 10 µm. **b** Quantification of Olig2 nuclear immunofluorescent (IF) signal. *n* = 4 independent biological samples. **c** Upregulated expression of p21/CDKN1A in GBM and DIPG stem-like cells (10 mM GHB, 1 week, Western blot assay, mean ± SD, *n* = 3 independent biological samples). p21 MW, 21 kDa; Actin MW, 42 kDa. RDU relative densitometry units. **d** GHB inhibits self-renewal of GBM (TG1) and DIPG (TP54) stem-like cells. Mean ± SD, *n* = 3 independent biological samples. **e** GHB inhibits cell growth of stem-like cells isolated from adult GBM and pediatric DIPG. Total numbers of viable cell evaluated after 1-week 10 mM GHB treatment. Mean ± SD, *n* = 3 independent biological samples. **f**, **g** GHB arrests cell cycle. **f** Example of cell cycle FACS analysis of GHB-treated TG1 GBM stem-like cells. BrdU labels cells having undergone DNA replication. DAPI is an index of DNA content. **g** Quantification of the cell cycle analyses. Mean ± SD, *n* = 3 independent biological samples. **h** *ALDH5A1* downregulation in GBM (TG1) and DIPG (TP54) stem-like cells inhibits cell proliferation. siA (*ALDH5A1* siRNA) versus siC (control siRNA), mean ± SD, *n* = 3 independent biological samples

To further probe the coherence between the in vitro findings and the observations in the patient tumors, we assayed GHB effects on GBM and DIPG cells focusing first on the expression of the transcription factor Olig2 and proliferation. These assays were performed while maintaining the cells in stem-media containing the EGF and bFGF mitogens. GHB inhibited the expression of Olig2 (Fig. 2a, b). Olig2 is required for the proliferation and tumorigenicity of adult GBM cells through repression of the inhibitor of cell cycle progression p21/CDKN1A [35]. Accordingly, Western blotting showed up-regulated p21 levels in GHB-treated cells (Fig. 2c), and upon siRNA-mediated *ALDH5A1* downregulation (Fig. S6A).

Inhibition of the self-renewal properties of adult GBM cells (TG1, 6240**, R633) and infant DIPG cells (TP54, TP83) accompanied these changes, as determined through the generation of secondary and tertiary spheres from single cells derived from primary spheres (Fig. 2d) or extreme limiting dilution assays (Fig. S6B). In presence of GHB, the percentage of cells yielding secondary and tertiary spheres dropped from 75–90 to 3–7, and 0–3%, respectively (Fig. 2d). In coherence with these findings, GHB inhibited proliferation of all 7 adult GBM and 4 infant DIPG cells tested. Cell numbers were reduced by 40–70% (Fig. 2e), while cell survival was unaffected (Fig. S6C and D). Cell cycle analysis of TG1 and TP54 cells showed a two- to threefold reduction in the number of cells in S phase (Fig. 2f, g), further confirming GHB inhibitory effects on cancer stem-like cell proliferation. Consistently, enhanced GHB production due to siRNA-mediated *ALDH5A1* downregulation in adult GBM (TG1) and infant DIPG (TP54) stem-like cells was accompanied by reduced cell proliferation similar to GHB treatment alone (Fig. 2h). Reduced proliferation rates were also observed in cells transduced with sh*ALDH5A1* compared to shControl (Fig. S6E). Furthermore, enhanced GHB production resulting from valproate inhibition of SSADH activity also resulted in robust inhibition of cell proliferation (Fig. S6F).

### SSADH-driven GHB accumulation induces GBM and DIPG stem-like cell differentiation into less aggressive cells

**Fig. 3:**
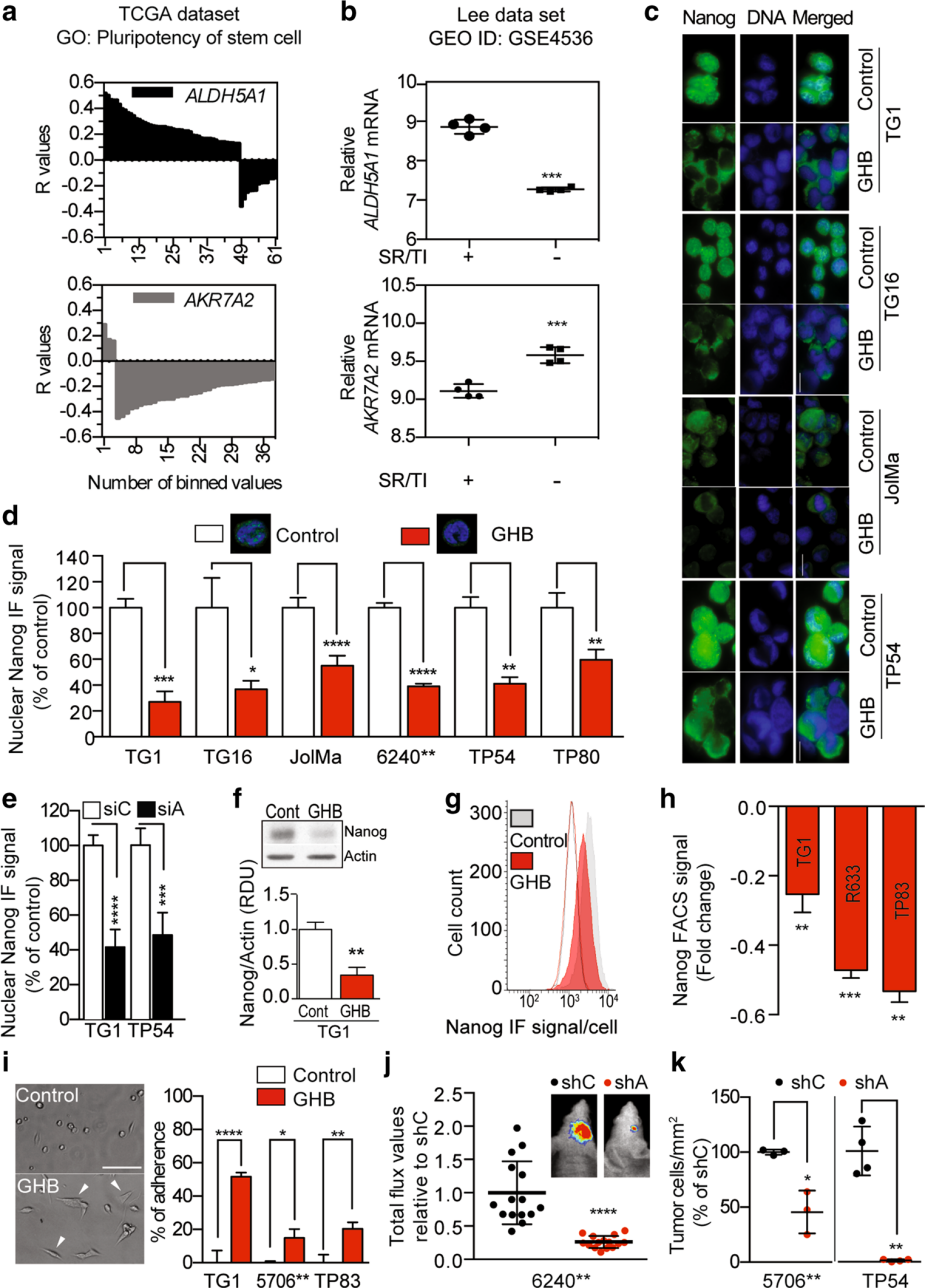
GHB promotes GBM and DIPG stem-like cell differentiation and decreases tumor burden. **a** Positive and negative correlation of *ALDH5A1* and *AKR7A2* expression with stemness genes (*p* < 0.01 after correction for multiple testing with false discovery rate). Gene ontology (GO) analysis using transcriptomes of 484 untreated primary human GBM of the TCGA dataset. Of the 142 listed in KEGG pathway category «Signaling pathways regulating pluripotency of stem cells» , 122 were detected in the dataset. 61 were significantly correlated with *ALDH5A1* (48 positive/13 negative) and 38 with *AKR7A2* (3 positive/35 negative). **b** Opposite regulation of *ALDH5A1* and *AKR7A2* transcript levels in cells with and without self-renewing and tumor-initiating properties (SR/TI) isolated from 4 human GBM [32]. Mean ± SD. **c**–**h** GHB inhibits Nanog expression. **c** Examples of immunocytochemical detection of Nanog. 1-week GHB 10 mM. *Scale bars* 10 µm. (**d**) Quantification of nuclear Nanog immunofluorescent (IF) signal. Mean ± SD, *n* = 3 independent biological samples. Microphotographs illustrate 240 nm single optical sections of TG1 cells immunostained for Nanog. **e** Decreased Nanog expression in response to *ALDH5A1* downregulation. siA, *ALDH5A1* siRNA. siC, control siRNA. Mean ± SD, *n* = 4 independent biological samples. **f** Nanog Western blot assay with protein extracts from control (Cont) and 10 mM GHB-treated TG1 GBM stem-like cells (1-week treatment). Nanog MW, 47 kDa; Actin MW, 42 kDa. *n* = 3 independent biological samples. RDU, relative densitometry units. **g** FACS analysis of Nanog immunofluorescent signal. *Grey* and *red lines* delineate unstained TG1 cells in control and GHB-treated conditions, respectively. **h** Fold change of mean fluorescent intensity of Nanog signal per cell (control vs 1 week-GHB 10 mM), as determined by FACS. TG1 and R633 GBM stem-like cells. TP83 DIPG stem-like cells. Mean ± SD, *n* = 4 independent biological samples. **i** GHB promotes cell adherence and membrane extension. Microphotograph of TG1 GBM stem-like treated with 10 mM GHB for 24 h and quantification of the numbers of adhering cells. Mean ± SD, *n* = 6 independent biological samples (TG1), *n* = 3 (5706**), *n* = 3 (TP83). *Scale bar* 100 µm. **j** Bioluminescent analyses of tumor growth initiated by grafting 6240** GBM stem-like cells transduced with a luciferase construct and a control (shC) or *ALDH5A1* (shA) shRNA construct. 49 days post-graft. Quantification of the bioluminescent signals. Mean ± SD, *n* = 15 mice per group. Photographs correspond to examples of bioluminescent in vivo images of tumors in mice. **k** Quantification of tumor cells numbers following xenografts of GFP-expressing 5706** GBM or TP54 DIPG stem-like cells transduced with a control or *ALDH5A1* shRNA construct. Mice were killed at 64 (5706**) or 71 (TP54) days post-graft, and the numbers of GFP-expressing cells determined. Mean ± SD, *n* = 3 (5706**) and 4 mice (TP54) per group

We further evaluated the relevance of our findings for the patient tumors by using independent dataset to probe links between SSADH expression and stem cell-like features that have been associated with glioma cell tumorigenicity [9]. The analysis of the TCGA transcriptome dataset of 484 untreated primary GBM was performed using the R2 Genomics Analysis and Visualization Platform (http://r2.amc.nl). The analysis disclosed a correlation positive for *ALDH5A1*, and negative for *AKR7A2* (encoding SSAR), with a majority of genes belonging to the KEGG pathway «Signaling pathways regulating pluripotency of stem cells» (Fig. 3a). 122 of the 142 genes listed in this category were detected in the dataset. 61 were significantly correlated with *ALDH5A1* (48 positive/13 negative) and 38 with *AKR7A2* (3 positive/35 negative). The same correlations were also found with the R2 platform’ gene category named «cancer gene census only» that contains 487 cancer-related genes (Fig S7A). Of the 371 cancer-related genes detected in the TCGA dataset, 190 were significantly correlated with *ALDH5A1* (130 positive/60 negative) and 178 with *AKR7A2* (23 positive/155 negative). In addition, the analysis of published transcriptome dataset of early-passage (P3) GBM cells either devoid of or endowed with self-renewing and tumor-initiating properties [32], revealed downregulated *ALDH5A1*, and conversely up-regulated *AKR7A2* expression in non-tumorigenic cells compared to tumorigenic cells (Fig. 3b). Using serum addition as well as growth factor removal as another means to promote cell exit from the stem state, we also observed downregulated *ALDH5A1* (Fig. S7B and C). These results led us to determine GHB effects on the expression of the stem transcription factor Nanog, which controls the stem-like and tumorigenic properties of GBM cells [34, 39, 59]. Immunofluorescent imaging showed a robust decrease in nuclear Nanog signal in GHB-treated GBM cells with either mesenchymal (TG1, TG16), or classical profiles (JolMa, 6240**) as well as in infant DIPG cells with pro-neural profiles (TP54, TP80) (Fig. 3c, d). Similar to GHB treatment, siRNA-mediated *ALDH5A1* downregulation in TG1 (adult GBM) and TP54 (infant DIPG) cells decreased Nanog nuclear localization (Fig. 3e). Decreased Nanog expression was confirmed by Western blotting (Fig. 3f, TG1), and FACS analysis (Fig. 3g, h, TG1, R633, TP83). We further found that GHB stimulated GBM stem-like cell adhesion on permissive substrates with formation of membrane extensions (Fig. 3i), and increased numbers of cells expressing the differentiation markers ß3-Tubulin and MAP2, or GFAP and EAAT2/GLT1 upon transfer in pro-neuronal or pro-astroglial differentiation media, respectively (Fig. S7D). In a coherent manner, *ALDH5A1* downregulation with shRNA, or valproate inhibition of SSADH activity were also accompanied with increased expression of differentiation markers (Fig. S7E and F).

Altogether, these results showed that SSADH-driven GHB accumulation repressed proliferation, clonality and stem-like features of malignant glioma cells. We therefore determined whether these repressive effects affected in vivo tumor development using orthotopic xenografts of GBM (6240**, 5706**) and DIPG (TP54) stem-like cells stably expressing either shControl or sh*ALDH5A1* (Fig. S8). GBM stem-like cells 6240**, which stably express luciferase, were used for bioluminescent imaging. Results showed reduced tumor burden in mice grafted with 6240**-sh*ALDH5A1* compared to mice grafted with 6240**-shControl cells (Fig. 3j). In addition, a significant improvement in survival of the mice grafted with 6240**-sh*ALDH5A1* cells was observed (Kaplan–Meier analysis, Fig. S9A). Decreased tumor burden was also observed with grafts of 5706**-sh*ALDH5A1* and TP54-sh*ALDH5A1*, as shown by cell counting (Figs. 3k, S9B).

### Inhibition of TET activity mediates GHB repressive effects on GBM and DIPG stem-like cells

GHB is naturally present in the brain at low concentrations and acts as an inhibitory neuromodulator through the GABA receptor GABA_B_R [12], and a GABA_A_R sub-class [1]. Using TG1 cells, we found that agonists of GABA_A_R (Isoguvacine hydrochloride) or GABA_B_R (Baclofen) did not modify GBM cell proliferation whereas GABA_A_R or GABA_B_R antagonists (Gabazine and Hydroxysaclofen) did not prevent GHB inhibition of cell proliferation (Fig. S10). GHB action being independent from GABA receptors, we investigated an intracellular mode of action.

**Fig. 4:**
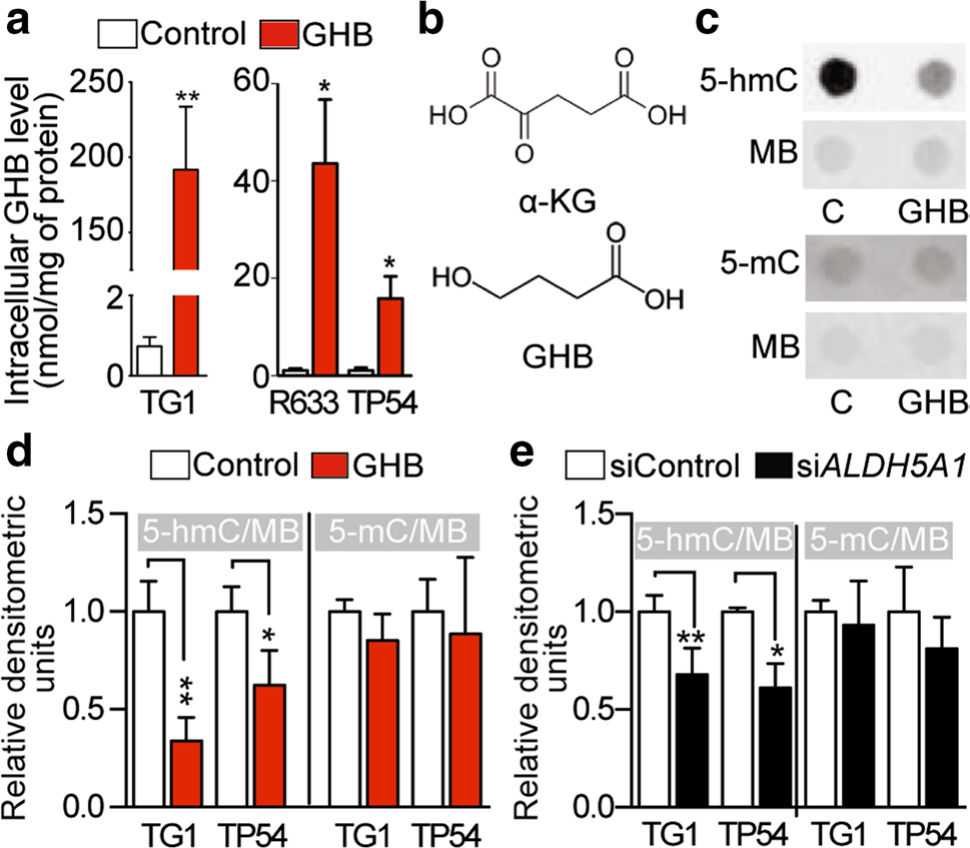
GHB accumulates in GBM and DIPG stem-like cells and decreases levels of the 5-hydroxymethylcytosine epigenetic mark. **a** Increased intracellular GHB levels detected by mass spectrometry following GHB supplementation (*n* = 3 independent biological samples). **b** Chemical structure of α-KG and GHB. **c** Example of 5-hmC and 5-mC detection by dot immunoblotting of DNA extracts from control (C) and GHB-treated GBM stem-like cells (TG1). Methylene blue (MB) was used as a loading control. **d** GHB decreases 5-hmC levels in GBM (TG1) and DIPG (TP54) stem-like cells without changing 5-mC levels. Densitometric analysis of dot immunoblotting of DNA extracts (10 mM GHB, 1 week). Mean ± SD, *n* = 3 (TG1) and *n* = 4 (TP54) independent biological samples. **e** *ALDH5A1* downregulation in GBM (TG1) or DIPG (TP54) stem-like cells results in decreased 5-hmC levels in face of unchanged 5-mC levels. Mean ± SD, *n* = 4 (TG1) and *n* = 3 (TP54) independent biological samples

GC–MS/MS analysis showed that GHB treatment led to increase in its intra-cellular levels (Fig. 4a). GHB penetration inside the cells, and the similarity of its chemical structure to that of α-KG (Fig. 4b), suggested that GHB could target α-KG-dependent enzymes such as Ten–eleven Translocations (TETs). These nuclear enzymes are needed for ESC maintenance in the stem state, and for reprogramming somatic cells into induced pluripotent stem cell (iPSC) [11, 14, 17]. They notably act by oxidizing 5-methylcytosine (5-mC) into 5-hmC [17], which has been shown recently to recruit a methylosome complex needed for glioblastomagenesis [51].

The evaluation of mRNA levels showed TET2 to be the most abundant of the three TET isoforms in the cells studied here (Fig. S11A). Dot immunoblotting of DNA extracts showed a marked reduction in 5-hmC levels in GHB-treated GBM (TG1) as in DIPG stem-like cells (TP54) compared to control conditions, whereas 5-mC levels were unchanged (Fig. 4c, d). Similar results were obtained using si*ALDH5A1*-transfected TG1 and TP54 cells (Fig. 4e). Compared to TG1, we also found reduced 5-hmC levels in TG1-miR (Fig. S11B) and in TG1 treated with serum (Fig. S11C), a well-known inducer of GBM cell differentiation into less tumorigenic cells. Notably, GHB did not alter 5-hmC levels in human fetal neural stem cells (NSC) (Fig. S11D).

**Fig. 5:**
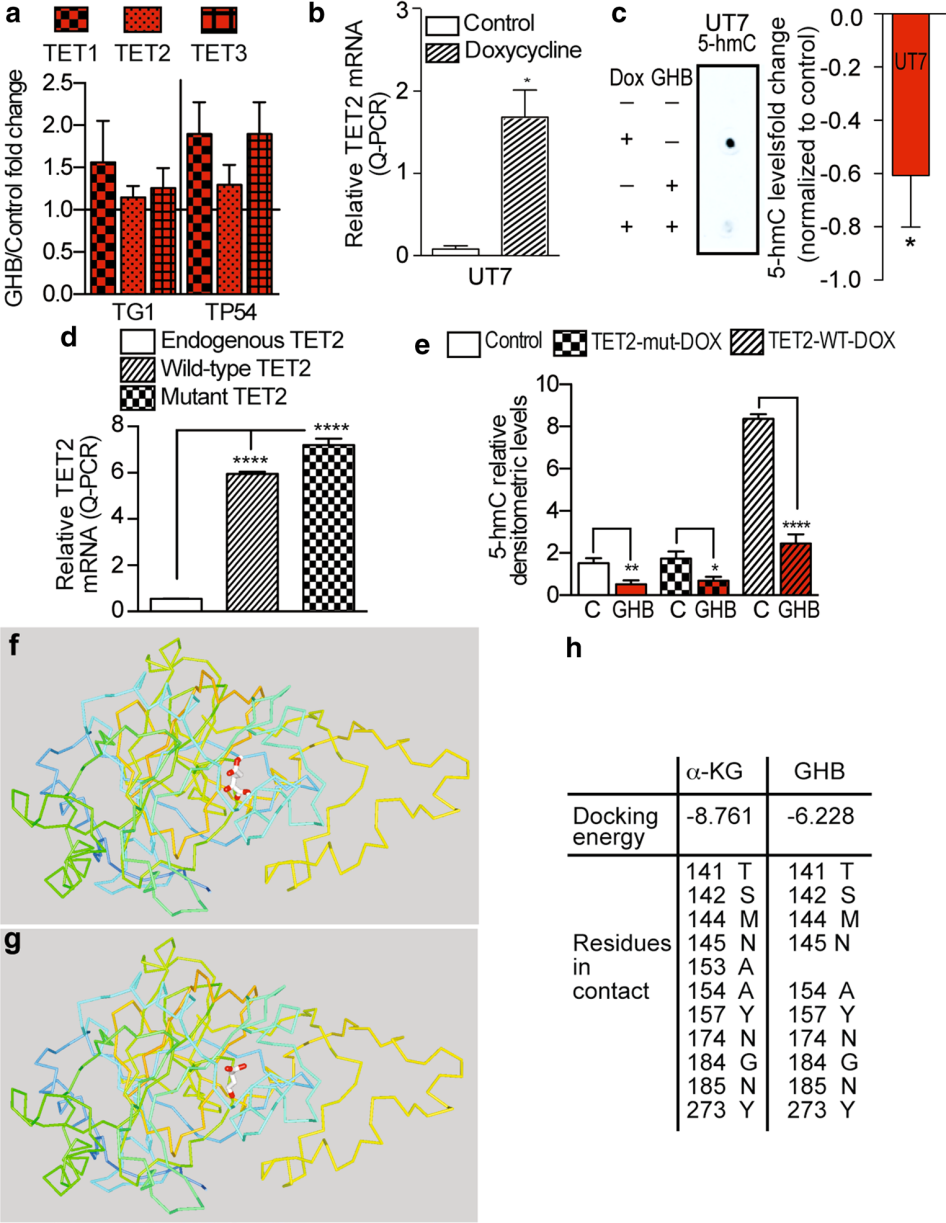
GHB decreases 5-hydroxymethylcytosine levels via inhibition of TET2 activity. **a** GHB does not reduce expression of TET isoforms. Q-PCR assay. Mean ± SD, *n* = 4 (TG1) and 3 (TP54) independent biological samples. **b** Doxycycline-dependent TET2 expression in the leukemic UT7 cell line. UT7 cells were treated for 12 h with doxycycline (Dox) at a final concentration of 1 µg/mL. TET2 mRNA levels were assayed using Q-PCR. Dox-treated versus -untreated UT7, mean ± SD, *n* = 3 independent biological samples. **c** GHB inhibits TET2 activity in the leukemic UT7 cell line expressing TET2 in a doxycycline-dependent manner. 12 h 10 mM GHB. 5-hmC levels determined by DNA dot immunoblotting. GHB-treated vs control UT7, mean ± SD, *n* = 3 independent biological samples. **d** Doxycycline-dependent TET2 expression in GBM stem-like cells (TG1) stably overexpressing TET2 in a doxycycline-dependent manner following lentiviral transduction. Q-PCR assays, mean ± SD, *n* = 5 biological samples. **e** Inhibitory GHB effects on TET2-mediated 5-hmC formation in GMB stem-like TG1 cells overexpressing TET2 in a doxycycline-dependent manner, mean ± SD, *n* = 4 biological samples. **f**–**h** In silico analysis of α-KG (**f**) and GHB (**g**) binding pockets within the TET2 protein. α-KG and GHB are colored in *white* and their oxygens in *red*. Ribbon representation of TET2 C-terminal domain. Progression from the N- to C- parts of the C-terminal domain are colored from *blue* to *orange*. **h** TET2 amino acid residues in contact with α-KG or GHB

Since GHB did not inhibit expression of TET2, or of the other TET isoforms (Fig. 5a), we pursued the study of GHB effect on TET2 enzymatic activity. To further confirm GHB inhibition of TET2 activity, we first used a UT7 leukemic cell line stably overexpressing TET2 in a doxycycline-dependent manner (Fig. 5b). In UT7 cells, 5-hmC was only detected upon induction of TET2 expression, and GHB strongly reduced 5-hmC levels (Fig. 5c). The same results were obtained with TG1 cells engineered to express either a doxycycline-inducible wild-type or a catalytically defective TET2 (Fig. 5d). Enhanced 5-hmC formation was only observed upon induction of wild-type TET2 expression, and was counteracted in presence of GHB (Fig. 5e). The analysis of TET2 structure confirmed the presence of a GHB binding pocket within TET2. As previously mentioned, α-KG is a co-factor required for TET2 oxidative activity. The crystal structure of TET2 has shown localization of α-KG binding site in the C-terminal domain of the protein [24]. To identify the site of GHB binding pocket in TET2, we performed in silico docking analysis on the GalaxyWEB platform (http://galaxy.seoklab.org/softwares/galaxydock.html [22]). We first confirmed the existence of a unique binding pocket for α-KG, formed by 11 amino acids located as expected in TET2 C-terminal domain (Fig. 5f). A unique binding pocket was also found for GHB (Fig. 5g), almost identical to the α-KG binding pocket: of the 11 amino acids forming the α-KG binding pocket, only 153A was not included in the GHB binding pocket (Fig. 5h). Calculated docking energies were similar for GHB and α-KG (−6.228 and −8.761 kcal/mole, respectively).

**Fig. 6:**
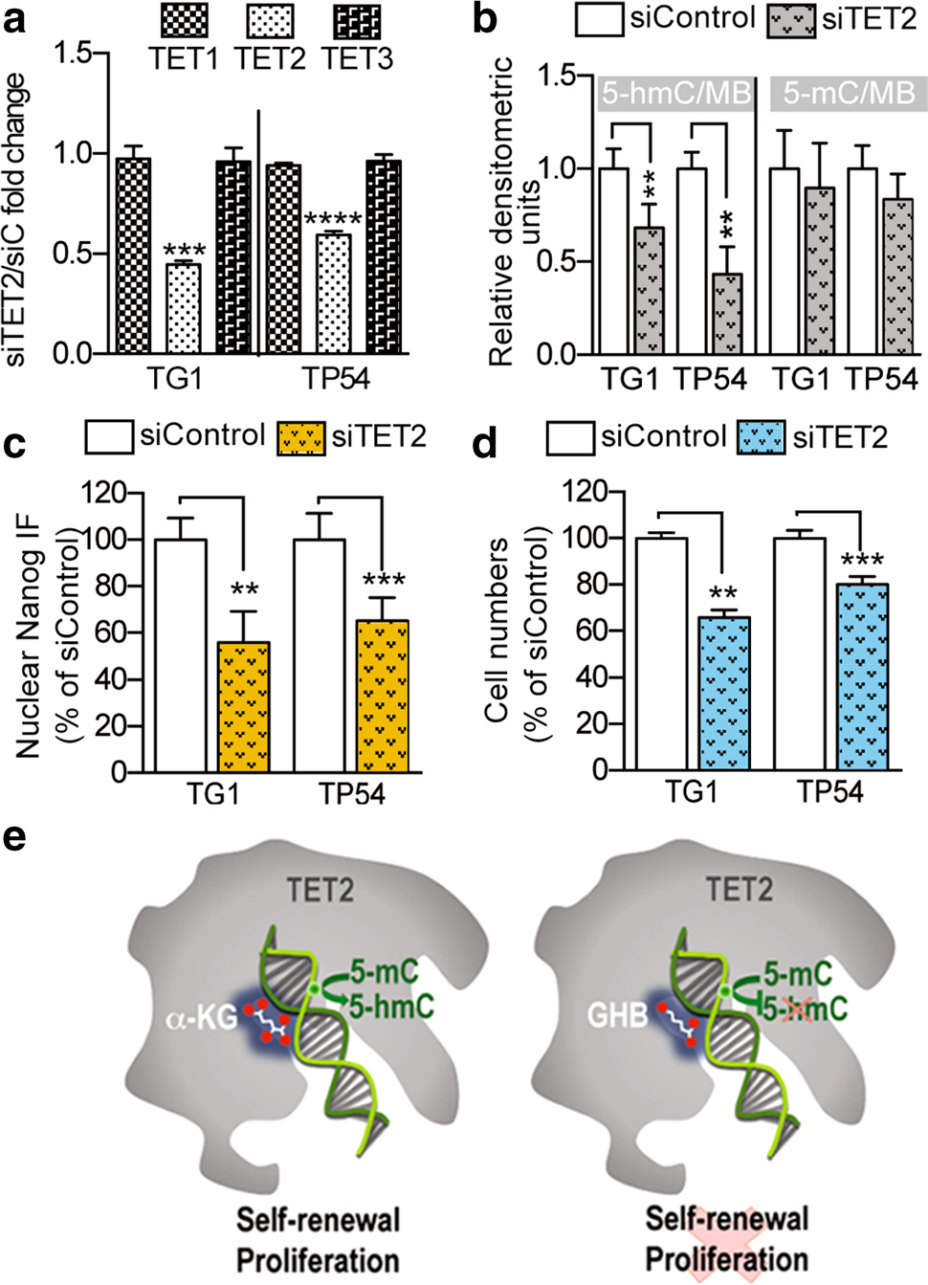
Biological effects of TET2 downregulation are comparable to GHB treatment or *ALDH5A1* downregulation. **a** TET2 downregulation using siRNA (siTET2) without changes in TET1 or TET3 mRNA levels. Q-PCR assay. Mean ± SD, *n* = 3 independent biological samples. siC, control siRNA. **b**–**d** TET2 downregulation translates into decreased 5-hmC levels (**b**), decreased Nanog immunofluorescent (IF) nuclear signal (**c**), and decreased proliferation (**d**). TET2 siRNA vs control siRNA, mean ± SD, *n* = 4 independent biological samples. **e** Schematic representation of GHB mechanism of action

Finally, to ascertain further the link between TETs and GHB, we used siRNA to downregulate TET2 (Fig. 6a). TG1 and TP54 cells were used as examples of GBM and DIPG cells, since GHB had the same effects on all the cells we had examined. TET2 downregulation had effects comparable to GHB treatment or *ALDH5A1* downregulation, i.e., reduced 5-hmC levels without significant change in 5-mC levels (Fig. 6b), decreased Nanog nuclear signals (Fig. 6c) and reduced cell proliferation (Fig. 6d).

Altogether, these results showed that GHB decreases 5-hmC levels by antagonizing TET activity, and that TET2 participates in the mechanisms by which GHB induces GBM and DIPG cell differentiation into less aggressive cells (Fig. 6e).

## Discussion

Understanding the molecular mechanisms underlying the genesis of heterogeneous cancer cell populations in brain tumors is likely to have profound impacts on therapeutic management. Here, we provide evidence that variations in expression of a single metabolic enzyme support cancer cell variegation by promoting GBM stem-like cell differentiation, hence favoring the genesis of cancer cell sub-populations with alleviated aggressiveness.

We show that elevating GHB intra-cellular levels through GHB supplementation, SSADH downregulation with si or shRNA or its pharmacological inhibition with valproate induces differentiation of stem-like cells isolated from distinct GBM and DIPG with differing profiles and genomic alterations. GHB inhibited cell self-renewal, proliferation, and expression of stem markers by repressing TET-mediated formation of 5-hmC. Analysis of transcriptome datasets from independent cohort of GBM tissues (TCGA) or cells [32] further associated high *ALDH5A1* expression with stem-like and cancerous behaviors within the context of GBM. Importantly, we disclosed strikingly distinct SSADH expression over GBM territories distinguished notably by their contents in proliferating cells. SSADH was enriched in non-differentiated, proliferative GBM territories compared to differentiated, weakly proliferative territories, which had scarce SSADH expression. These differing SSADH expression patterns were associated with elevated GHB/α-KG ratios in differentiated territories. These results, associated with reduced tumor-initiating properties of GBM cells with repressed SSADH expression in orthotopic xenograft assays, support the functional relevance of GABA metabolism reprogramming in the context of the patient tumors. In addition, the increase in GHB extra-cellular levels accompanying increased GHB production, suggests that this GABA by-product may promote through a paracrine mode of action the genesis of tumor territories enriched in weakly proliferative cells. Brain cancer stem-like cells are sensitive to environmental cues, and epigenetic regulators are known translators of extra-cellular cues. Binding sites for factors such as YY1, which can interact with major epigenetic regulators such as histone methyltransferases [47] and thus translate extra-cellular cues, are present in *ALDH5A1* gene regulatory regions (Ensembl platform, ENSG00000112294). The miR-302 can be another relevant epigenetic regulator, since it targets *ALDH5A1* mRNA, is upregulated in GBM stem-like cells by extra-cellular pro-differentiation cues [15], and is enriched in non-proliferative/differentiated GBM territories (F. Burel-Vandenbos, T. Virolle, unpublished results). Another level of SSADH regulation that might be relevant within a tumor context is its sensitivity to oxidation. Biochemical studies showed that oxidative stress inactivates SSADH through the formation of disulfide bonds between cysteine residues of the catalytic domain, the enzyme being reactivated upon environmental switch to reducing conditions [30]. SSADH expression appears, thus, likely to be sensitive to variations in the cell environment, but the identity of such extra-cellular cues remains to be identified.

Valproate repression of SSADH activity can also be relevant in a tumor context. First used as an anti-epileptic agent, this pharmacological compound has been found to repress proliferation and promote differentiation of different cancer cell types and notably of glioblastoma cells [19, 23]. To our knowledge, we provide the first demonstration that valproate increases GHB levels in the context of cancer. Valproate anti-cancerous action is usually attributed to its inhibitory effect on histone deacetylases. Our results suggest that valproate anti-cancerous effects could be mediated at least in part by increased GHB production. This therefore adds to the current debate of the potential use of valproate as an adjuvant treatment for glioblastoma patients [23, 44, 56].

GHB, like GABA, is first known to act through its binding to GABA receptors [1, 12]. GABA itself has been reported to inhibit ESC and NSC proliferation through GABA_A_R activation [3, 16]. Our results with GABAR agonists and antagonists indicate that the restraint exerted by GABA on NSC or ESC cycling is absent in GBM stem-like cells. Unchanged 5-hmC levels in GHB-treated NSC further supports the idea that GHB repressive effects on GBM stem-like cell behavior depends on an intra-cellular mode of action targeting TET.

TET hydroxylation of methyl groups on cytosine was first interpreted as an intermediate step in the process of DNA demethylation [17] through passive replication-dependent demethylation [27] or active enzymatic modification of the nucleotide [28]. GHB-induced decrease in 5-hmC formation did not change global DNA methylation levels. This observation is consistent with the recent recognition of 5-hmC as an epigenetic mark per se [50], notably in the context of pro-neural GBM [51]. This metabolic control of GBM stem-like cells is akin to that recently disclosed in ESC. Maintenance of high ratios of α-KG over TCA metabolites, which might interfere with its role as an enzyme cofactor to support TET and additional epigenetic modifiers activities, was shown to be essential for preventing ESC differentiation [8]. Although GHB effects on other α-KG dependent epigenetic modifiers cannot be excluded, our results demonstrate that TET activity is crucial not only for GBM stem-like cells with profiles resembling mesenchymal or classical GBM subtypes [55] but also for stem-like cells isolated from DIPG. These systematically fatal tumors are constituted of cells with abundant 5-hmC content distributed through the pons [2], and bear a H3F3.A K27M mutation [48, 58] that prevents enzymatic activity of the Polycomb repressive complex 2 [6, 33]. Our results show that GHB repressive effects on TET activity can take place in a context of major deregulation of chromatin modifiers.

Finding that TET inhibition underpins GHB off-target anti-tumor effect in GBM and DIPG stem-like cells stretches out further the variable outcome of TET activity according to the cancer type considered. *TET2* loss-of-function mutations were identified in myeloid and lymphoid hematological malignancies [13], whereas the *TET1* overexpression found in mixed lineage leukemia (MLL)-rearranged leukemia is oncogenic [26]. Context dependency of TET (dys)function in cancer is further indicated by lack of correlation between 5-hmC levels and IDH1/2 mutations in slow growing infiltrative gliomas, although these mutations drive overproduction of the 2-HG metabolite that may interfere with α-KG actions as a co-factor [31, 38].

Altogether, our results reveal that switching GABA catabolism toward GHB production opposes the proliferation and stem-like properties of GBM and DIPG cells. They also show that a neuromodulator can directly interfere with an epigenetic modifier. These GHB repressive effects are consistent with suppression of GBM cell tumorigenic properties upon SSADH downregulation as well as finding elevated GHB/α-KG ratios with scarce SSADH expression in tumor territories characterized by low numbers of proliferating cells and lack of Olig2 expression. We, thus, uncover a metabolic state with a driving role in repressing aggressiveness of cancer cells in malignant glioma. Although no specific SSADH pharmacological inhibitor is currently available, the ability of GHB to cross the blood brain barrier and its approved use for several indications (e.g., narcolepsy, alcohol withdrawal) [12, 29, 36], suggest that it could be effective to target cancer cells dependent on 5-hmC formation for their growth.

## Electronic supplementary material

Below is the link to the electronic supplementary material.

Supplementary material 1 (PDF 6448 kb)

Supplementary material 2 (XLSX 78 kb)

Supplementary material 3 (XLSX 48 kb)

## References

[1] Absalom N, Eghorn LF, Villumsen IS, Karim N, Bay T, Olsen JV, Knudsen GM, Brauner-Osborne H, Frolund B, Clausen RP (2012). alpha4betadelta GABA(A) receptors are high-affinity targets for gamma-hydroxybutyric acid (GHB). Proc Natl Acad Sci USA.

[2] Ahsan S, Raabe EH, Haffner MC, Vaghasia A, Warren KE, Quezado M, Ballester LY, Nazarian J, Eberhart CG, Rodriguez FJ (2014). Increased 5-hydroxymethylcytosine and decreased 5-methylcytosine are indicators of global epigenetic dysregulation in diffuse intrinsic pontine glioma. Acta neuropathol Commun.

[3] Andang M, Hjerling-Leffler J, Moliner A, Lundgren TK, Castelo-Branco G, Nanou E, Pozas E, Bryja V, Halliez S, Nishimaru H (2008). Histone H2AX-dependent GABA(A) receptor regulation of stem cell proliferation. Nature.

[4] Assad Kahn S, Costa SL, Gholamin S, Nitta RT, Dubois LG, Feve M, Zeniou M, Coelho PLC, El-Habr E, Cadusseau J (2016). The anti-hypertensive drug prazosin inhibits glioblastoma growth via the PKCdelta-dependent inhibition of the AKT pathway. EMBO Mol Med.

[5] Auffinger B, Tobias AL, Han Y, Lee G, Guo D, Dey M, Lesniak MS, Ahmed AU (2014). Conversion of differentiated cancer cells into cancer stem-like cells in a glioblastoma model after primary chemotherapy. Cell Death Differ.

[6] Bender S, Tang Y, Lindroth AM, Hovestadt V, Jones DT, Kool M, Zapatka M, Northcott PA, Sturm D, Wang W (2013). Reduced H3K27me3 and DNA hypomethylation are major drivers of gene expression in K27 M mutant pediatric high-grade gliomas. Cancer Cell.

[7] Brocard E, Oizel K, Lalier L, Pecqueur C, Paris F, Vallette FM, Oliver L (2015). Radiation-induced PGE2 sustains human glioma cells growth and survival through EGF signaling. Oncotarget.

[8] Carey BW, Finley LW, Cross JR, Allis CD, Thompson CB (2015). Intracellular alpha-ketoglutarate maintains the pluripotency of embryonic stem cells. Nature.

[9] Chen J, McKay RM, Parada LF (2012). Malignant glioma: lessons from genomics, mouse models, and stem cells. Cell.

[10] Chiesa-Vottero AG, Rybicki LA, Prayson RA (2003). Comparison of proliferation indices in glioblastoma multiforme by whole tissue section vs tissue microarray. Am J Clin Pathol.

[11] Costa Y, Ding J, Theunissen TW, Faiola F, Hore TA, Shliaha PV, Fidalgo M, Saunders A, Lawrence M, Dietmann S (2013). NANOG-dependent function of TET1 and TET2 in establishment of pluripotency. Nature.

[12] Crunelli V, Emri Z, Leresche N (2006). Unravelling the brain targets of gamma-hydroxybutyric acid. Curr Opin Pharmacol.

[13] Delhommeau F, Dupont S, Della Valle V, James C, Trannoy S, Masse A, Kosmider O, Le Couedic JP, Robert F, Alberdi A (2009). Mutation in TET2 in myeloid cancers. N Engl J Med.

[14] Doege CA, Inoue K, Yamashita T, Rhee DB, Travis S, Fujita R, Guarnieri P, Bhagat G, Vanti WB, Shih A (2012). Early-stage epigenetic modification during somatic cell reprogramming by Parp1 and Tet2. Nature.

[15] Fareh M, Turchi L, Virolle V, Debruyne D, Almairac F, de-la-Forest Divonne S, Paquis P, Preynat-Seauve O, Krause KH, Chneiweiss H et al (2012) The miR 302-367 cluster drastically affects self-renewal and infiltration properties of glioma-initiating cells through CXCR4 repression and consequent disruption of the SHH-GLI-NANOG network. Cell Death Diff 19:232–244. doi:10.1038/cdd.2011.89 10.1038/cdd.2011.89 PMC3263498 21720384

[16] Fernando RN, Eleuteri B, Abdelhady S, Nussenzweig A, Andang M, Ernfors P (2011). Cell cycle restriction by histone H2AX limits proliferation of adult neural stem cells. Proc Natl Acad Sci USA.

[17] Ficz G, Branco MR, Seisenberger S, Santos F, Krueger F, Hore TA, Marques CJ, Andrews S, Reik W (2011). Dynamic regulation of 5-hydroxymethylcytosine in mouse ES cells and during differentiation. Nature.

[18] Flavahan WA, Wu Q, Hitomi M, Rahim N, Kim Y, Sloan AE, Weil RJ, Nakano I, Sarkaria JN, Stringer BW (2013). Brain tumor initiating cells adapt to restricted nutrition through preferential glucose uptake. Nat Neurosci.

[19] Gottlicher M, Minucci S, Zhu P, Kramer OH, Schimpf A, Giavara S, Sleeman JP, Lo Coco F, Nervi C, Pelicci PG (2001). Valproic acid defines a novel class of HDAC inhibitors inducing differentiation of transformed cells. EMBO J.

[20] Gupta PB, Fillmore CM, Jiang G, Shapira SD, Tao K, Kuperwasser C, Lander ES (2011). Stochastic state transitions give rise to phenotypic equilibrium in populations of cancer cells. Cell.

[21] Hanahan D, Weinberg RA (2011). Hallmarks of cancer: the next generation. Cell.

[22] Heo L, Lee H, Baek M, Seok C (2016). Binding site prediction of proteins with organic compounds or peptides using GALAXY web servers. Methods Mol Biol (Clifton, NJ).

[23] Hoja S, Schulze M, Rehli M, Proescholdt M, Herold-Mende CH, Riemenschneider MJ (2016). Molecular dissection of the valproic acid effects on glioma cells. Oncotarget.

[24] Hu L, Li Z, Cheng J, Rao Q, Gong W, Liu M, Shi YG, Zhu J, Wang P, Xu Y (2013). Crystal structure of TET2-DNA complex: insight into TET-mediated 5mC oxidation. Cell.

[25] Hu Y, Smyth GK (2009). ELDA: extreme limiting dilution analysis for comparing depleted and enriched populations in stem cell and other assays. J Immunol Methods.

[26] Huang H, Jiang X, Li Z, Li Y, Song CX, He C, Sun M, Chen P, Gurbuxani S, Wang J (2013). TET1 plays an essential oncogenic role in MLL-rearranged leukemia. Proc Natl Acad Sci USA.

[27] Inoue A, Zhang Y (2011). Replication-dependent loss of 5-hydroxymethylcytosine in mouse preimplantation embryos. Science.

[28] Ito S, Shen L, Dai Q, Wu SC, Collins LB, Swenberg JA, He C, Zhang Y (2011). Tet proteins can convert 5-methylcytosine to 5-formylcytosine and 5-carboxylcytosine. Science.

[29] Keating GM (2014). Sodium oxybate: a review of its use in alcohol withdrawal syndrome and in the maintenance of abstinence in alcohol dependence. Clin Drug Investig.

[30] Kim Y-G, Lee S, Kwon O-S, Park S-Y, Lee S-J, Park B-J, Kim K-J (2009). Redox-switch modulation of human SSADH by dynamic catalytic loop. EMBO J.

[31] Kraus TF, Globisch D, Wagner M, Eigenbrod S, Widmann D, Munzel M, Muller M, Pfaffeneder T, Hackner B, Feiden W (2012). Low values of 5-hydroxymethylcytosine (5hmC), the “sixth base,” are associated with anaplasia in human brain tumors. Int J Cancer Journal international du cancer.

[32] Lee J, Kotliarova S, Kotliarov Y, Li A, Su Q, Donin NM, Pastorino S, Purow BW, Christopher N, Zhang W (2006). Tumor stem cells derived from glioblastomas cultured in bFGF and EGF more closely mirror the phenotype and genotype of primary tumors than do serum-cultured cell lines. Cancer Cell.

[33] Lewis PW, Muller MM, Koletsky MS, Cordero F, Lin S, Banaszynski LA, Garcia BA, Muir TW, Becher OJ, Allis CD (2013). Inhibition of PRC2 activity by a gain-of-function H3 mutation found in pediatric glioblastoma. Science.

[34] Li Y, Li A, Glas M, Lal B, Ying M, Sang Y, Xia S, Trageser D, Guerrero-Cazares H, Eberhart CG (2011). c-Met signaling induces a reprogramming network and supports the glioblastoma stem-like phenotype. Proc Natl Acad Sci USA.

[35] Ligon KL, Huillard E, Mehta S, Kesari S, Liu H, Alberta JA, Bachoo RM, Kane M, Louis DN, Depinho RA (2007). Olig2-regulated lineage-restricted pathway controls replication competence in neural stem cells and malignant glioma. Neuron.

[36] Mayer G (2012). The use of sodium oxybate to treat narcolepsy. Expert Rev Neurother.

[37] Meyer M, Reimand J, Lan X, Head R, Zhu X, Kushida M, Bayani J, Pressey JC, Lionel AC, Clarke ID (2015). Single cell-derived clonal analysis of human glioblastoma links functional and genomic heterogeneity. Proc Natl Acad Sci USA.

[38] Muller T, Gessi M, Waha A, Isselstein LJ, Luxen D, Freihoff D, Freihoff J, Becker A, Simon M, Hammes J (2012). Nuclear exclusion of TET1 is associated with loss of 5-hydroxymethylcytosine in IDH1 wild-type gliomas. Am J Pathol.

[39] Natsume A, Ito M, Katsushima K, Ohka F, Hatanaka A, Shinjo K, Sato S, Takahashi S, Ishikawa Y, Takeuchi I (2013). Chromatin regulator PRC2 is a key regulator of epigenetic plasticity in glioblastoma. Cancer Res.

[40] Parker NR, Khong P, Parkinson JF, Howell VM, Wheeler HR (2015). Molecular heterogeneity in glioblastoma: potential clinical implications. Front Oncol.

[41] Patel AN, Tirosh I, Trombetta JJ, Shalek AK, Gillespie SM, Wakimoto H, Cahill DP, Nahed BV, Curry WT, Martuza RL (2015). Single-cell RNA-seq highlights intratumoral heterogeneity in primary glioblastoma. Science.

[42] Patru C, Romao L, Varlet P, Coulombel L, Raponi E, Cadusseau J, Renault-Mihara F, Thirant C, Leonard N, Berhneim A (2010). CD133, CD15/SSEA-1, CD34 or side populations do not resume tumor-initiating properties of long-term cultured cancer stem cells from human malignant glio-neuronal tumors. BMC Cancer.

[43] Preusser M, de Ribaupierre S, Wohrer A, Erridge SC, Hegi M, Weller M, Stupp R (2011). Current concepts and management of glioblastoma. Ann Neurol.

[44] Redjal N, Reinshagen C, Le A, Walcott BP, McDonnell E, Dietrich J, Nahed BV (2016). Valproic acid, compared to other antiepileptic drugs, is associated with improved overall and progression-free survival in glioblastoma but worse outcome in grade II/III gliomas treated with temozolomide. J Neurooncol.

[45] Reitman ZJ, Jin G, Karoly ED, Spasojevic I, Yang J, Kinzler KW, He Y, Bigner DD, Vogelstein B, Yan H (2011). Profiling the effects of isocitrate dehydrogenase 1 and 2 mutations on the cellular metabolome. Proc Natl Acad Sci USA.

[46] Rosenberg S, Verreault M, Schmitt C, Guegan J, Guehennec J, Levasseur C, Marie Y, Bielle F, Mokhtari K, Hoang-Xuan K (2016). Multi-omics analysis of primary glioblastoma cell lines shows recapitulation of pivotal molecular features of parental tumors. Neuro-oncology.

[47] Satijn DP, Hamer KM, den Blaauwen J, Otte AP (2001). The polycomb group protein EED interacts with YY1, and both proteins induce neural tissue in Xenopus embryos. Mol Cell Biol.

[48] Schwartzentruber J, Korshunov A, Liu XY, Jones DT, Pfaff E, Jacob K, Sturm D, Fontebasso AM, Quang DA, Tonjes M (2012). Driver mutations in histone H3.3 and chromatin remodelling genes in paediatric glioblastoma. Nature.

[49] Silvestre DC, Pineda JR, Hoffschir F, Studler JM, Mouthon MA, Pflumio F, Junier MP, Chneiweiss H, Boussin FD (2011). Alternative lengthening of telomeres in human glioma stem cells. Stem Cells.

[50] Song CX, He C (2013). Potential functional roles of DNA demethylation intermediates. Trends Biochem Sci.

[51] Takai H, Masuda K, Sato T, Sakaguchi Y, Suzuki T, Suzuki T, Koyama-Nasu R, Nasu-Nishimura Y, Katou Y, Ogawa H (2014). 5-Hydroxymethylcytosine plays a critical role in glioblastomagenesis by recruiting the CHTOP-methylosome complex. Cell Rep.

[52] Thirant C, Bessette B, Varlet P, Puget S, Cadusseau J, Tavares Sdos R, Studler JM, Silvestre DC, Susini A, Villa C (2011). Clinical relevance of tumor cells with stem-like properties in pediatric brain tumors. PLoS One.

[53] Thirant C, Galan-Moya EM, Dubois LG, Pinte S, Chafey P, Broussard C, Varlet P, Devaux B, Soncin F, Gavard J (2012). Differential proteomic analysis of human glioblastoma and neural stem cells reveals HDGF as a novel angiogenic secreted factor. Stem Cells.

[54] van der Laan JW, de Boer T, Bruinvels J (1979). Di-n-propylacetate and GABA degradation. Preferential inhibition of succinic semialdehyde dehydrogenase and indirect inhibition of GABA-transaminase. J Neurochem.

[55] Verhaak RG, Hoadley KA, Purdom E, Wang V, Qi Y, Wilkerson MD, Miller CR, Ding L, Golub T, Mesirov JP (2010). Integrated genomic analysis identifies clinically relevant subtypes of glioblastoma characterized by abnormalities in PDGFRA, IDH1, EGFR, and NF1. Cancer Cell.

[56] Weller M (2013). Are we ready for a randomized trial of valproic acid in newly diagnosed glioblastoma?. Neuro-oncology.

[57] Whittle SR, Turner AJ (1981). Biogenic aldehyde metabolism in rat brain. Differential sensitivity of aldehyde reductase isoenzymes to sodium valproate. Biochim Biophys Acta.

[58] Wu G, Broniscer A, McEachron TA, Lu C, Paugh BS, Becksfort J, Qu C, Ding L, Huether R, Parker M (2012). Somatic histone H3 alterations in pediatric diffuse intrinsic pontine gliomas and non-brainstem glioblastomas. Nat Genet.

[59] Zbinden M, Duquet A, Lorente-Trigos A, Ngwabyt SN, Borges I, Ruiz IAA (2010). NANOG regulates glioma stem cells and is essential in vivo acting in a cross-functional network with GLI1 and p53. Embo J.

