# Supplementary material for: A driver role for GABA metabolism in controlling stem and proliferative cell state through GHB production in glioma
Source: Acta Neuropathol. 2016 Dec 28;133(4):645–60. doi: 10.1007/s00401-016-1659-5 (PMC5348560; doi:10.1007/s00401-016-1659-5)
Supplement: Supplementary file 1 — Supplementary material 1 (PDF 6448 kb) [file 401_2016_1659_MOESM1_ESM.pdf]

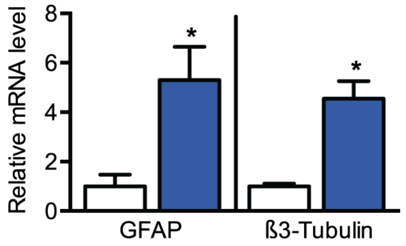

Figure S1. Expression of the differentiation markers ß3-Tubulin and GFAP in TG1-miR compared to TG1. Q-PCR assays. Mean±SD, n=3 independent biological samples.

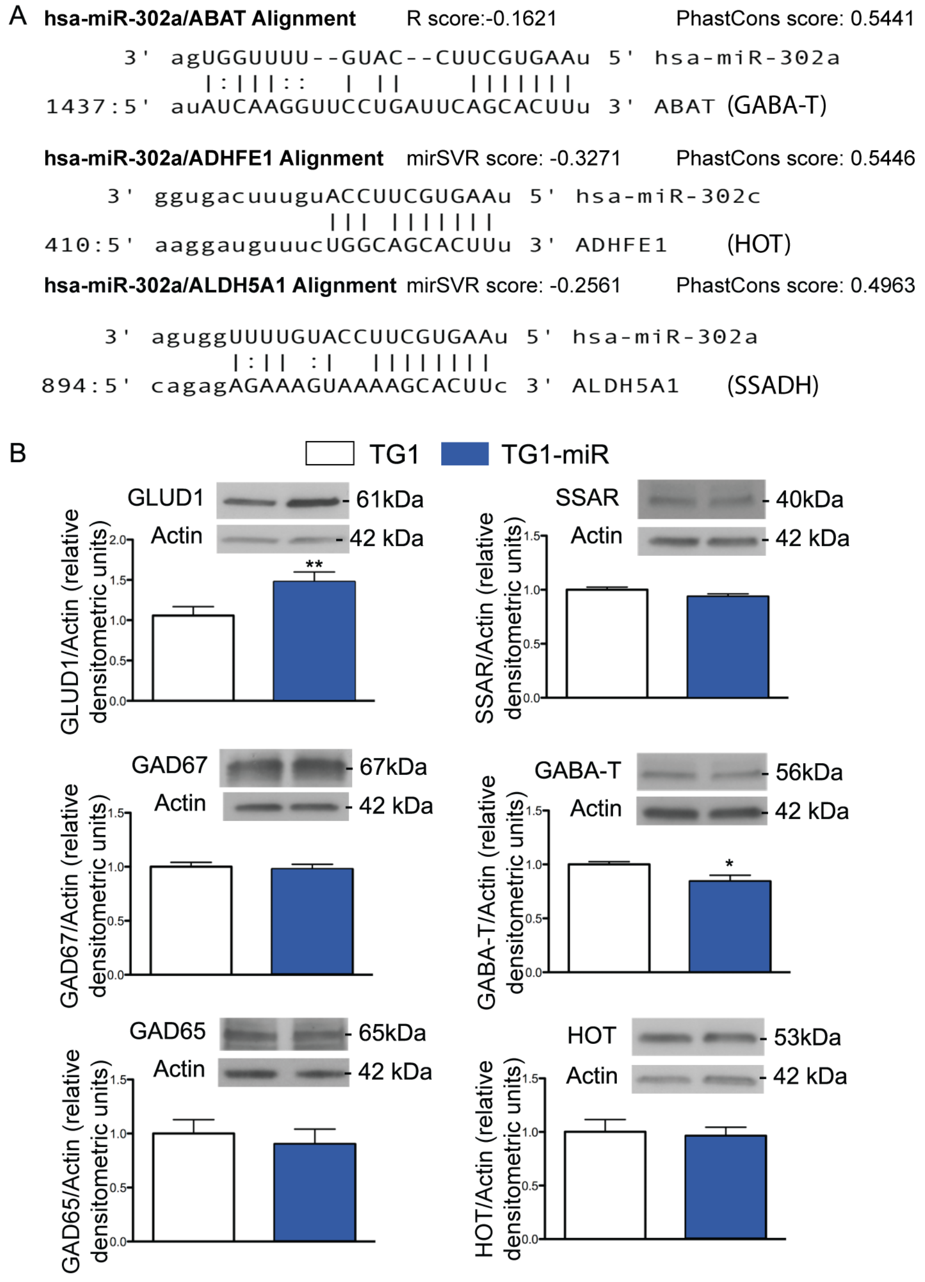

Figure S2. (A) In silico analysis of miR-302-367 putative targets (http://www.microrna.org/microrna/getGeneForm.do) identified putative miR-302 recognition sites in the mRNA 3'UTR of ABAT (coding for GABA-T), ADHFE1 (coding for HOT) and ALDH5A1 (coding for SSADH). (B) Expression of enzymatic components of the GABA metabolic pathway in TG1 and TG1-miR. The expression levels of the enzymes related to the metabolism of the GABA neurotransmitter were analyzed at the protein level by Western blot. Note the increase in GLUD1 protein levels and the decrease in GABA-T protein levels in TG1-miR compared to TG1. mean±SD, n=3-5 independent biological samples, *p<0.05, **p<0.01. See text for detailed description of the GABA metabolic pathway.

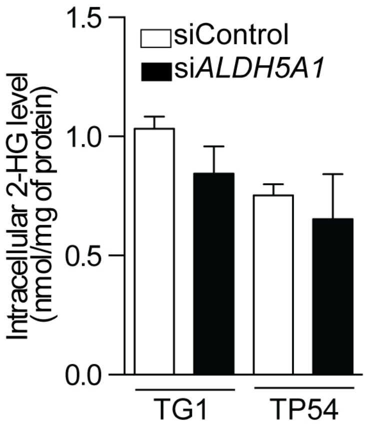

Figure S3. Unchanged intra-cellular levels of the other GABA by-product 2-HG upon ALDH5A1 down regulation. Mean ±SD, n=3 independent biological samples.

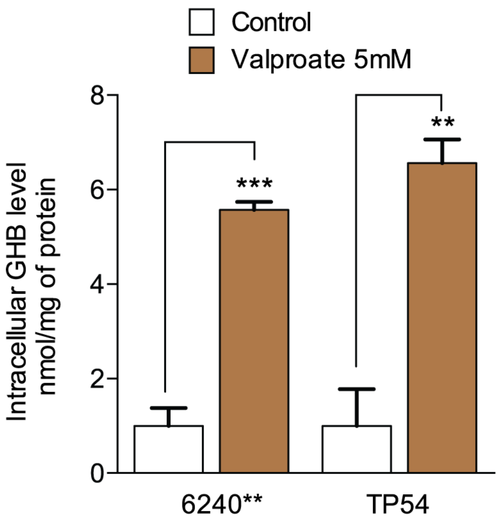

Figure S4. Increased intracellular GHB levels detected by mass spectrometry in cells treated for 7 days with 5mM valproate. Mean ±SD, n=3 independent biological samples.

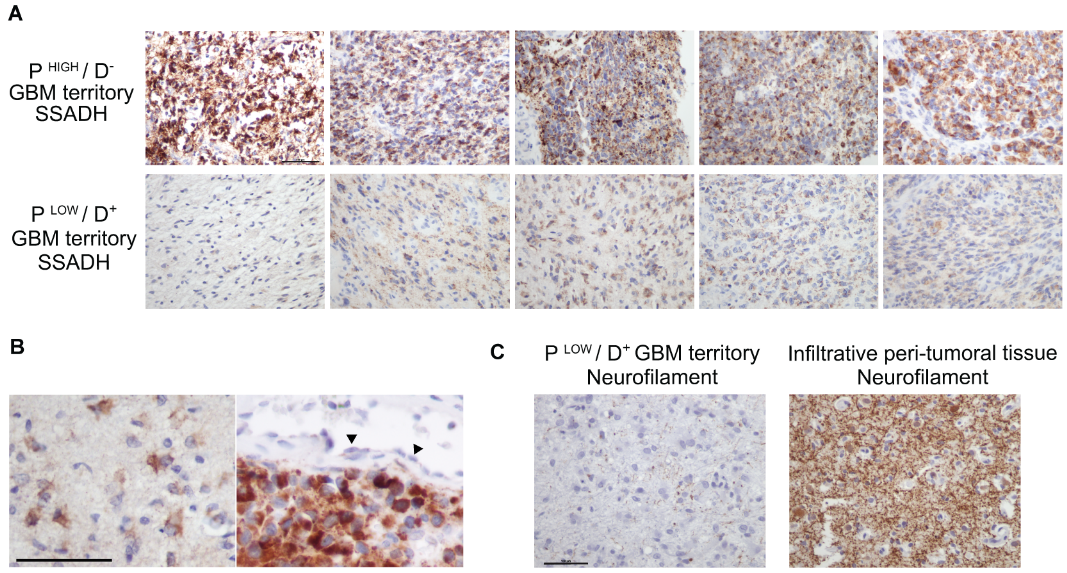

Figure S5. (A) Illustration of SSADH immunostaining in independent patients' GBM. SSADH immunoreactive cells are enriched in proliferative/non-differentiated GBM territories (P^{HIGH}/D^{-}) comparatively to non-proliferative/differentiated (P^{LOW}/D^{+}) tumor territories. Scale bar = 100µm. (B) SSADH immunostaining in normal astrocytes (left panel) and endothelial cells (right panel, arrowheads) compared to tumor cells of proliferative/non-differentiated GBM territories (right panel). Scale bar = 100µm. (C) Destruction of the axonal network in differentiated solid tumor tissues compared to infiltrative peri-tumoral tissue, as shown using neurofilament imunostaining.

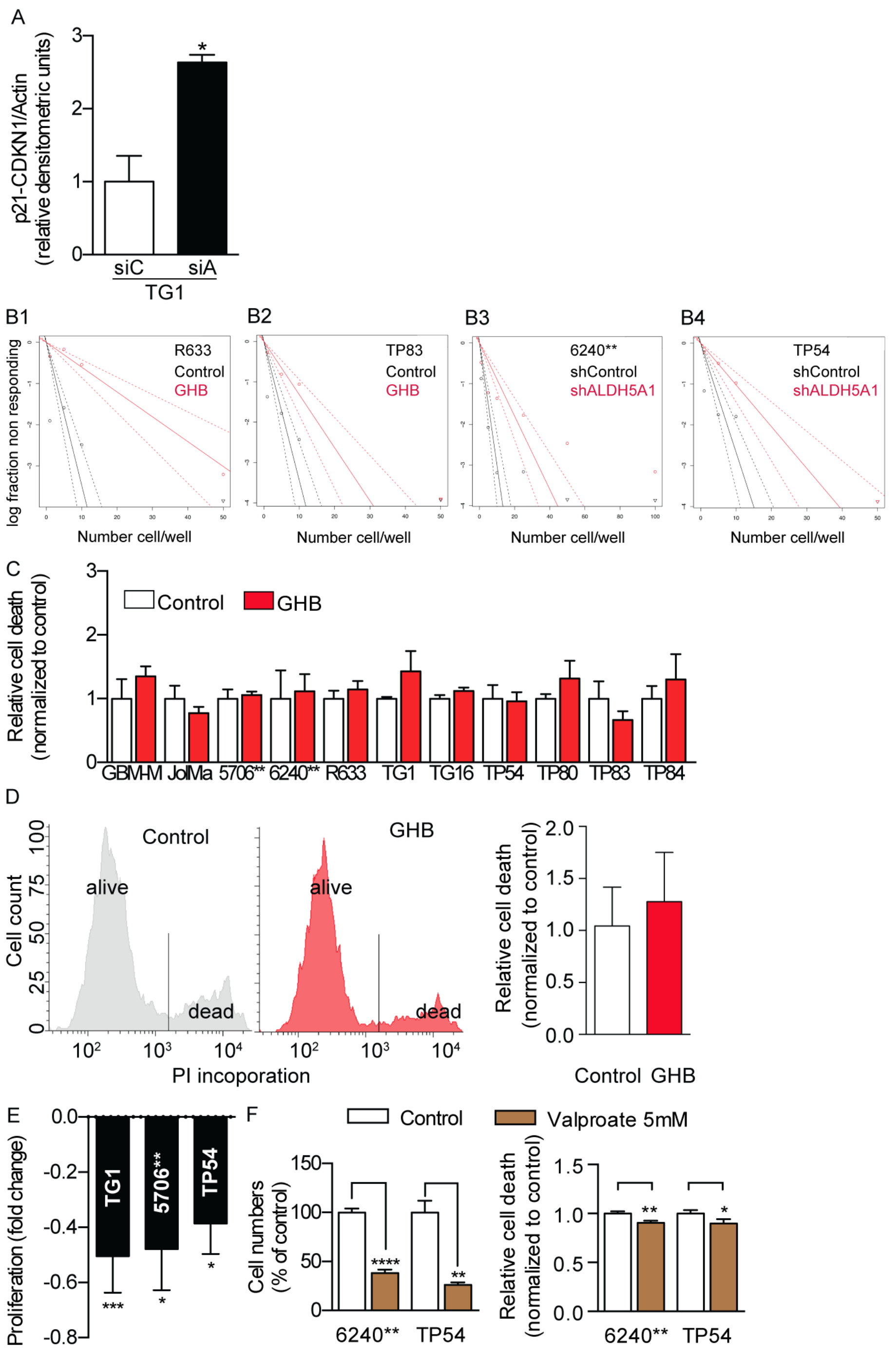

Figure S6. (A) Up-regulated p21 levels upon siRNA-mediated ALDH5A1 downregulation. Mean±SD, n=3 independent biological samples. (B1-B4) Extreme limiting dilution assays showing reduced sphere-forming capability upon GHB-treatment (B1-2) or ALDH5A1 downregulation with shRNA (B3-4). Sphere formation was scored 10 days post-seeding. Frequency of sphere-forming cells : R633 control 1/3.93 (lower 3.93, upper 2.17) vs GHB 1/16.6 (lower 23.66, upper 11.7), n=4, p=4.68 10−15. TP83 control = 1/2.91 (lower 3.99, upper 2.18) vs GHB 1/7.6 (lower 10.61, upper 5.49), n = 4, p = 6.98 10−6. 6240** shControl = 1/3.32 (lower 4.47, upper 2.51) vs shALDH5A1 1/11.12 (lower 14.79, upper 8.4), n = 6, p = 1.24 10−10. TP54 shControl = 1/3.74 (lower 5.12, upper 2.79) vs shALDH5A1 1/9.78 (lower 13.89, upper 6.93), n = 4, p = 2 10−5. (C-D) GHB does not alter survival of GBM and DIPG stem-like cells.
(C) Quantification of dead cell numbers as determined by Trypan blue exclusion assay, mean±SD, n=3 independent biological samplest. (D) Example of cell survival analysis (TG1) using FACS and propidium iodide incorporation. 1-week post-treatment with 10mM GHB (left panel), and the corresponding quantification of dead cell numbers (right panel, mean±SD, n=3 independent biological samples). (E) Reduced proliferation rates upon ALDH5A1 downregulation with shRNA. Mean±SD, n=6 (TG1) and n=3 (5706**, TP54) independent biological samples.
(F) Valproate inhibits cell proliferation (left panel) whereas it inhibits only marginally cell survival (right panel). 7 day-treatment. Mean±SD, n=3 independent biological samples.

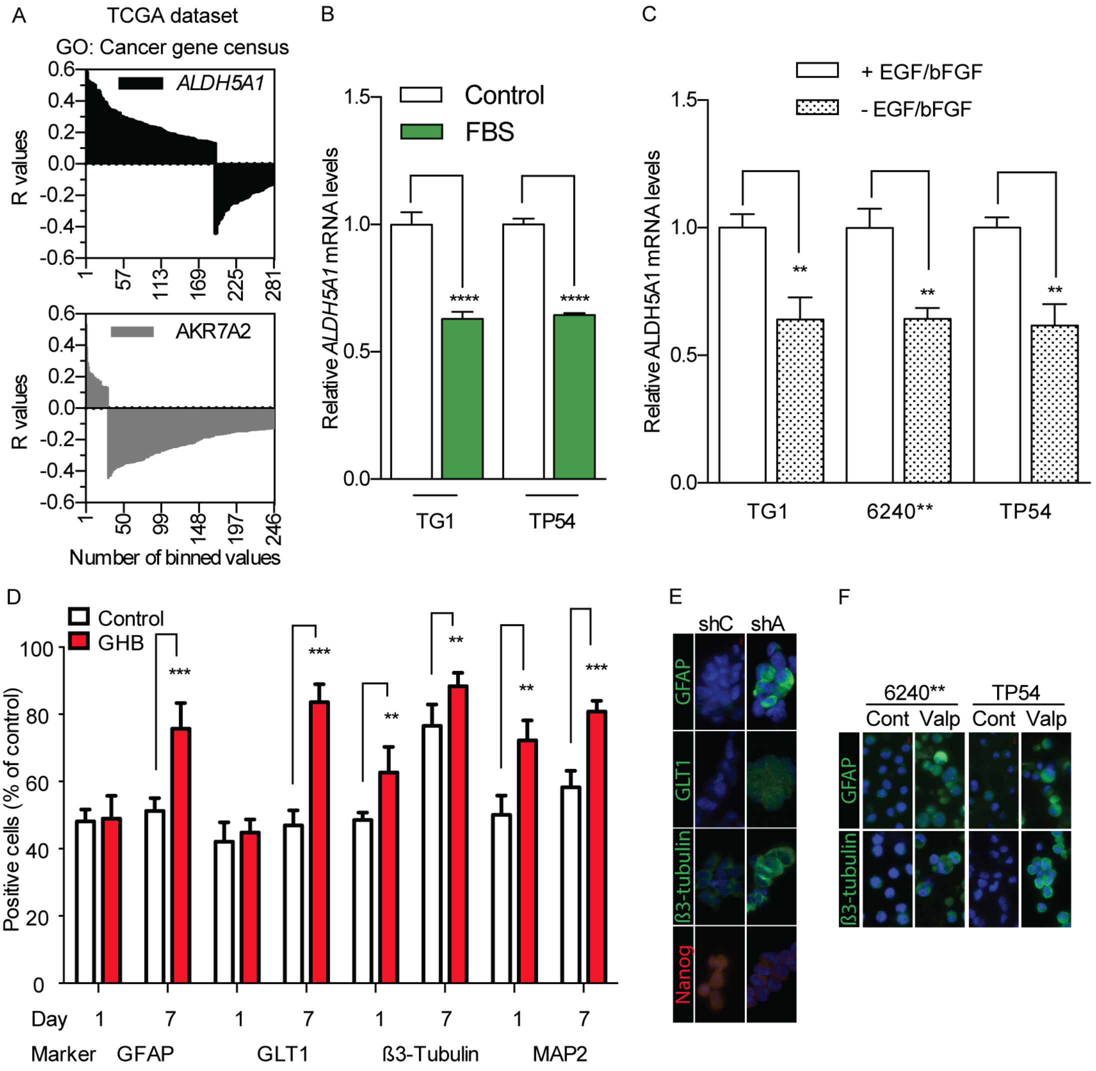

Figure S7. (A) Positive and negative correlation of ALDH5A1 and AKR7A2 expression with cancer-related genes ($p<0.01$ after correction for multiple testing with false discovery rate). Gene ontology (GO) analysis using transcriptomes of 484 untreated primary human GBM of the TCGA dataset. Detection in the dataset of 371 of the 487 cancer-related genes listed in the GO category R2 «cancer gene census only». 190 of them were significantly correlated with ALDH5A1 (130 positive/60 negative) and 178 with AKR7A2 (23 positive/155 negative). (B) Decreased ALDH5A1 mRNA levels in cells having exited the stem state following serum (FBS) addition. Mean±SD, n=3 independent biological samples. (C) Decreased ALDH5A1 mRNA levels in cells having exited the stem state following growth factor removal. Mean±SD, n=3 independent biological samples. (D) GHB increases the number of cells expressing the differentiation markers ß3-Tubulin and MAP2, or GFAP and EAAT2/GLT1 upon transfer of GBM stem-like cells (TG1) in pro-neuronal (Neurobasal supplemented with B27) or pro-astroglial (DMEM/F12 supplemented with 1% serum) differentiation media. Mean±SD, n=5 biological samples. (E) Increased ß3-Tubulin, GFAP and GLT1 expression, and concomitant decreased Nanog expression in 6240** stem-like cells expressing shALDH5A1 (shA) compared to shControl (shC). (F) Increased ß3-Tubulin and GFAP expression in valproate-treated cells (6240** and TP54). Cont: Control. Valp: Valproate.

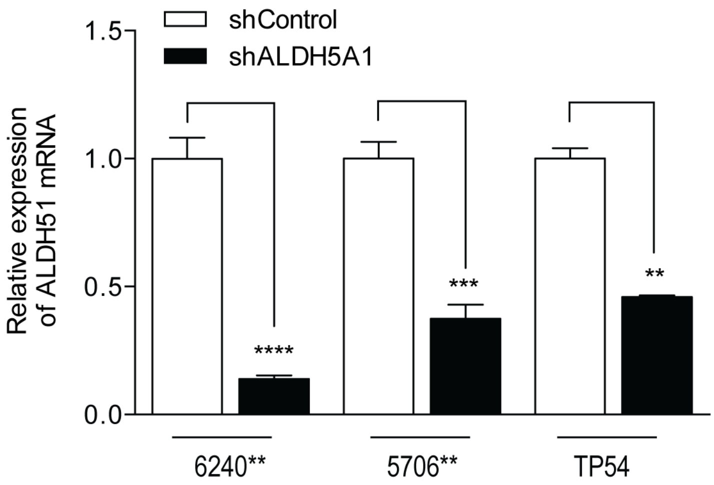

Figure S8. shRNA-driven downregulation of ALDH5A1 in GBM (6240**, 5706**) and DIPG (TP54) stem-like cells. Q-PCR assay, mean±SD, n=5 (6240**) and n=3 (5706**, TP54) independent biological samples.

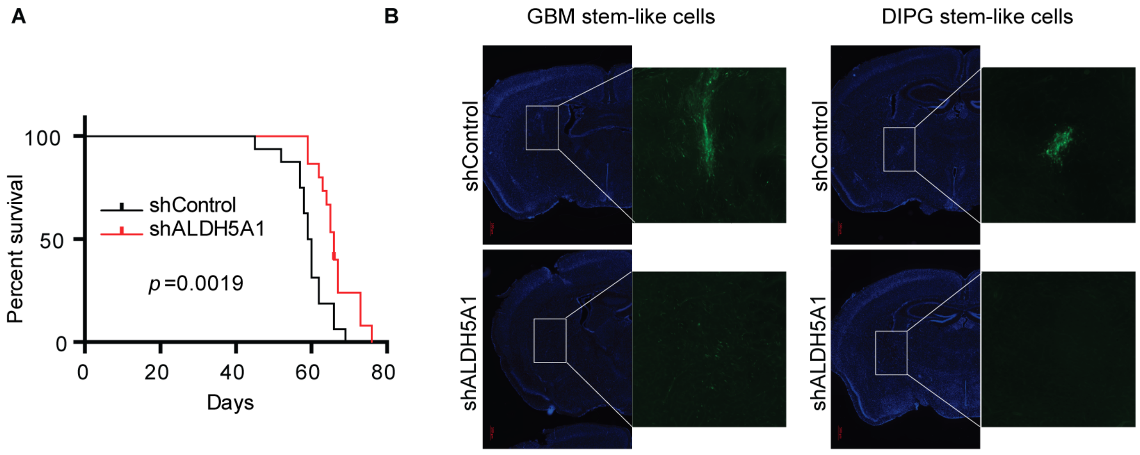

Figure S9. Orthotopic xenografts of GBM (6240**, 5706**) or DIPG (TP54) stem-like cells transduced with a control or ALDH5A1 shRNA construct. (A) Kaplan-Meyer survival curves demonstrating a significant survival benefit of mice grafted with 6240**-shALDH5A1 cells (15 mice) compared to mice grafted with 6240**-shControl cells (16 mice), log-rank Mantel-Cox test. (B) Illustration of GFP-expressing tumor cells in mice grafted with 5706** or TP54 expressing shControl or shALDH5A1. Mice were sacrificed at 64 (5706**) or 71 (TP54) days post-graft. Scale bar = 100 μM.

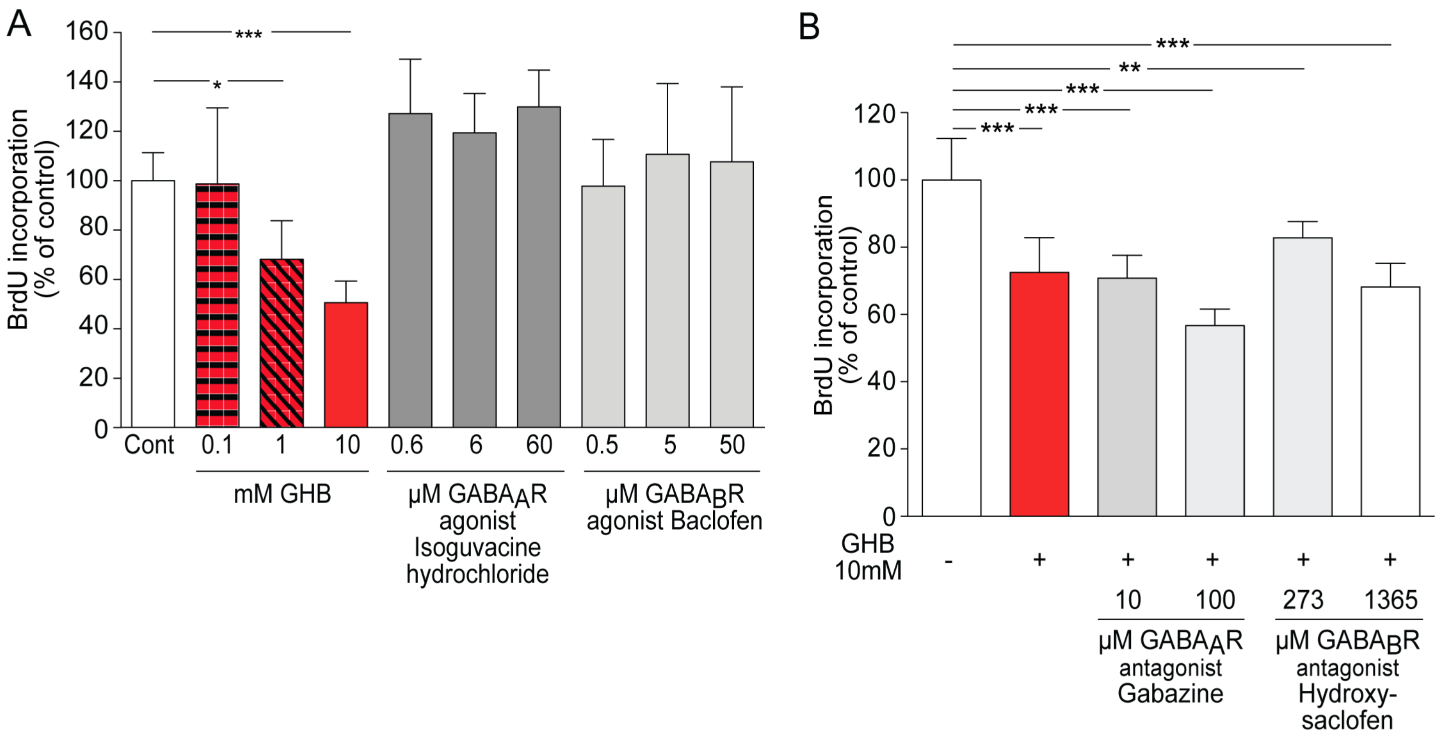

Figure S10. (A) The GABAAR agonist Isoguvacine hydrochloride, and the GABABR agonist Baclofen did not modify BrdU incorporation by GBM stem-like cells, whereas GHB decreased BrdU incorporation (TG1, 10mM GHB). Agonists were used at their reported EC50 concentration and at concentrations ten times less or more than their EC50. Mean±SD, n=3 biological samples. (B) The GABAAR antagonist Gabazine, or the GABABR antagonist Hydroxysaclofen did not prevent GHB inhibitory effect on BrdU incorporation (TG1, 10mM GHB). Antagonists were used at their reported EC50 concentration and at a concentration ten times more than their EC50. Mean±SD, n=3 biological samples.

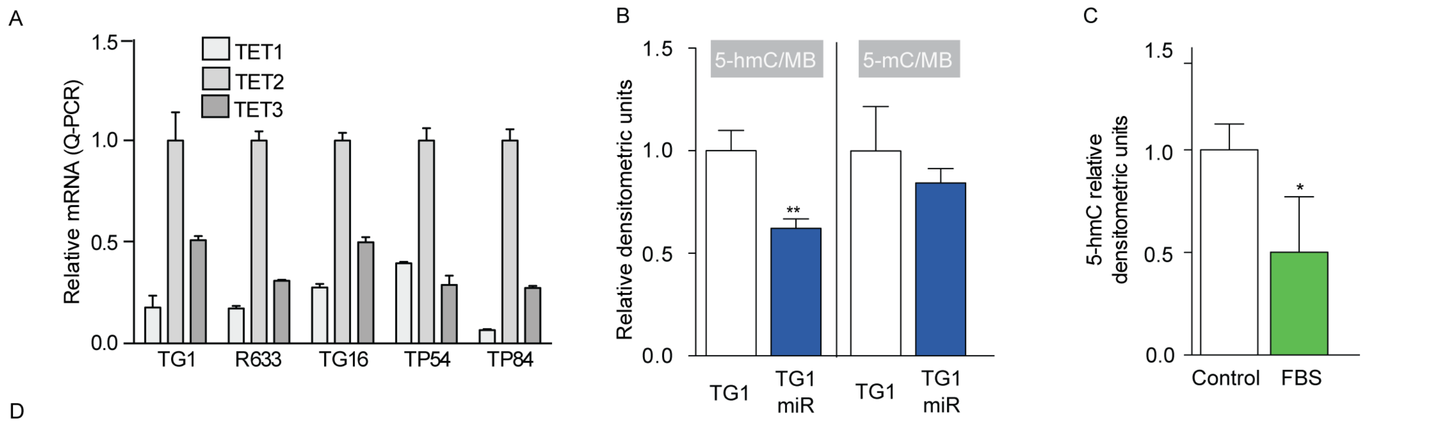

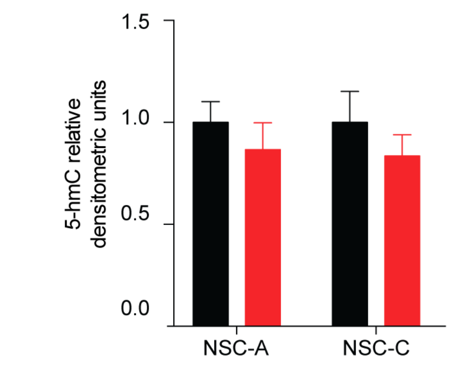

Figure S11. (A) Comparative TET1, TET2 and TET3 mRNA levels in GBM (TG1, TG16, R633) and DIPG (TP54, TP84) stem-like cells. Q-PCR assay. Mean±SD, n=3 independent biological samples. (B-C) Decreased 5-hmC levels accompany GBM cell exit from the stem state in response to either (B) miR-302-367 expression or (C) serum (FBS). Methylene blue (MB) was used as a loading control. (B) 5-hmC and 5-mC levels assayed using dot immunobloting of DNA extracts from TG1 and TG1-miR. Mean±SD, n=4 biological samples. (C) 5-hmC levels following TG1 exposure for 7 days to 2% FBS. Mean±SD, n=5 biological samples. (D) Unchanged 5-hmC and 5-mC levels in GHB-treated human fetal NSC. Mean±SD, n=4 biological samples.
